# Regioselectivity of Cobalamin‐Dependent Methyltransferase Can Be Tuned by Reaction Conditions and Substrate

**DOI:** 10.1002/cctc.202001296

**Published:** 2020-10-01

**Authors:** Simona Pompei, Christopher Grimm, Judith E. Farnberger, Lukas Schober, Wolfgang Kroutil

**Affiliations:** ^1^ Institute of Chemistry NAWI Graz University of Graz Heinrichstrasse 28 8010 Graz Austria; ^2^ Austrian Centre of Industrial Biotechnology c/o Institute of Chemistry University of Graz Heinrichstrasse 28 8010 Graz Austria; ^3^ Field of Excellence BioHealth University of Graz 8010 Graz Austria; ^4^ BioTechMed Graz 8010 Graz Austria

**Keywords:** regioselectivity, methylation, biocatalysis, cobalamin-dependent methyltransferases, medium engineering

## Abstract

Regioselective reactions represent a significant challenge for organic chemistry. Here the regioselective methylation of a single hydroxy group of 4‐substituted catechols was investigated employing the cobalamin‐dependent methyltransferase from *Desulfitobacterium hafniense*. Catechols substituted in position four were methylated either in *meta*‐ or *para*‐position to the substituent depending whether the substituent was polar or apolar. While the biocatalytic cobalamin dependent methylation was *meta*‐selective with 4‐substituted catechols bearing hydrophilic groups, it was *para*‐selective for hydrophobic substituents. Furthermore, the presence of water miscible co‐solvents had a clear improving influence, whereby THF turned out to enable the formation of a single regioisomer in selected cases. Finally, it was found that also the pH led to an enhancement of regioselectivity for the cases investigated.

## Graphical abstract

**Biocatalysis**: Regioselective reactions represent a challenge for organic chemistry. Here the regioselective mono‐methylation of 4‐substituted catechols was investigated employing the cobalamin‐dependent methyltransferase from *Desulfitobacterium hafniense*. The influence of polar and apolar substituents in position four, co‐solvents, as well as pH were examined. Water miscible co‐solvents allowed to tune the regioselectivity, whereby THF turned out to enable the formation of a single regioisomer in selected cases.

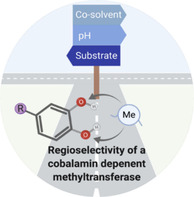

## Introduction

Controlling regioselectivity in a chemical transformation represents a challenge, since the differentiation between identical chemical groups within a single molecule can in general only be addressed by exploiting steric effects. Thus, the regioselectivity observed may be a result either of the specific properties of the substrate,^[1]^ or in a more general approach be controlled by the reagent or the catalyst. Consequently, beside metalorganic catalysts^[2]^ mainly biocatalysts^[3]^ have been reported for regioselective transformations like for instance in hydrolytic reactions,^[4]^ C−H oxidation,^[5]^ amination,^[6]^ Baeyer Villiger oxidation,^[7]^ and nitration,^[8]^ just to mention a few.

**Scheme 1:**
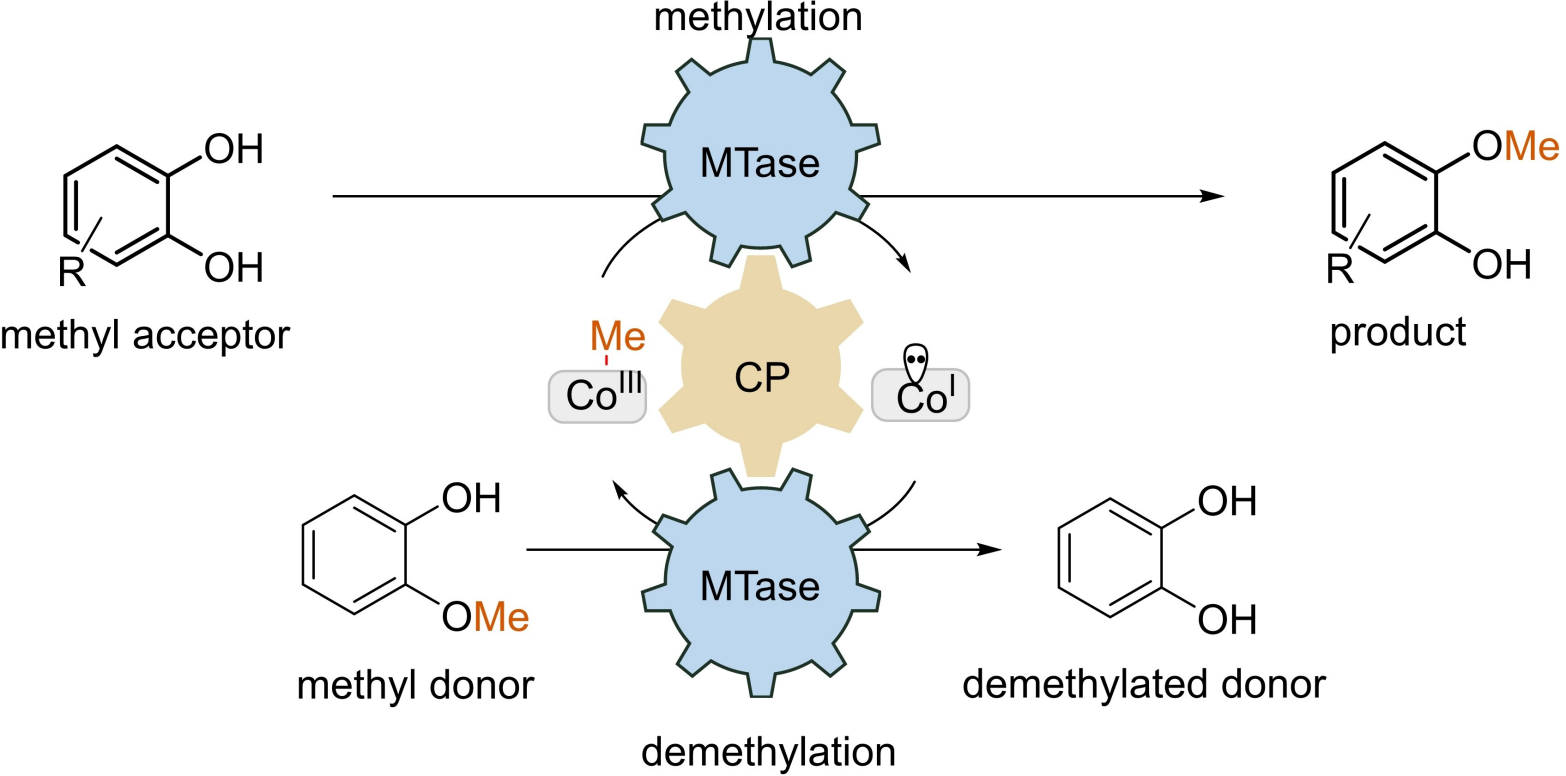
*o*‐Methylation coupled to a demethylation resulting in methyl transfer catalysed by the methyltransferase MTase I from *Desulfitobacterium hafniense*.

The methylation of catechols may give access to a variety of chemical products and intermediates such as flavoring agents, antioxidants and agrochemicals.^[9]^ Such aryl methyl ethers are synthesized chemically by *O*‐methylation of the corresponding phenols with monohalomethanes or dimethyl sulfate.^[10]^ Although various other methods have been established for their synthesis, like the use of dimethyl carbonate in the presence of metal catalysts,^[11]^ the regioselective methylation of catechols remains difficult. One biocatalytic approach for the methylation of catechols involves S‐adenosylmethionine (SAM) dependent methyltransferase which have great potential for selective alkylation processes.^[12]^ Yet, the enzymatic methylation offered by this class of enzymes is often hindered by the need for a stoichiometric supply of SAM cofactor and the inhibitory effect of the SAM‐derived byproduct on most methyltransferases.^[13]^ Nevertheless, recycling systems for SAM have been developed recently which will show their potential in the future.^[14]^ Beside the set of SAM‐dependent methyltransferases, corrinoid‐dependent methyltransferases (MTases)^[15]^ represent an alternative group of enzymes which may be considered for ether formation. Cobalamin‐dependent methyltransferases^[15]^ have recently been described for both, *O*‐methylation as well as demethylation (Scheme 1).^[16]^ The advantage of this class of enzyme is, that the methylated cofactor methylcobalamin can easily be recycled. The methyltransferase (MTase) catalyzes the transfer of the methyl group from a methyl donor to cobalamin bound to the corrinoid protein (CP); this reaction corresponds to a demethylation. The same MTase also catalyzes subsequently the transfer of the methyl group from methylcobalamin to the substrate/methyl acceptor. The MTase as well as the CP used here originate from the anaerobic bacteria *Desulfitobacterium hafniense* (MTase I). Although the reaction represents an equilibrium, the methylation can be shifted to the product side by using an excess of methyl donor.

Initial results^[16a]^ indicated, that the methylation of 4‐substituted catechols **1** lead exclusively to monomethylated products and may display a certain regioselectivity. Herein we investigate to which extend reaction conditions as well as the substrate influence the regioselectivity, thus how medium‐ and substrate engineering^[17]^ allow to modulate the formation of the one isomer over the other.

## Results and Discussion

**Scheme 2:**
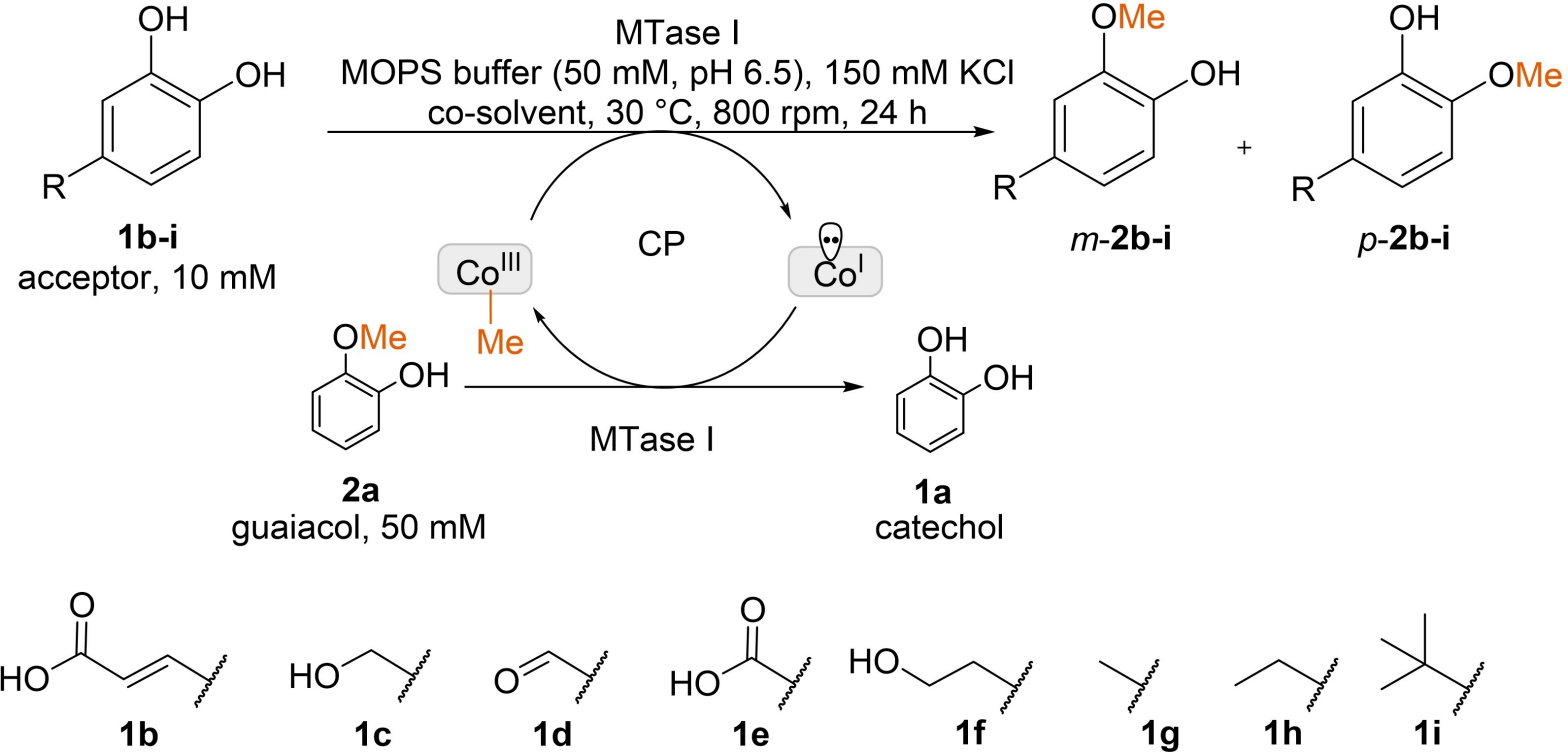
Methylation of 4‐substituted catechol derivatives **1 b**–**i** employing guaiacol **2 a** as methylating reagent. The *meta*‐ and *para* descriptors refer to the substituent R.

Regioselectivity may by altered or tuned by various options like by enzyme engineering^[18]^ or substrate and medium engineering.[^17^, ^19^] To learn about the influence of the reaction medium and the substituents on the regioselectivity of the MTase I for methylation of 4‐substituted catechols **1 b**–**i** (Scheme 2), three parameters were investigated: (i) the use of co‐solvents, (ii) the nature of the substituent in position 4 and (iii) the pH. For all the performed studies, guaiacol **2 a** was chosen as methyl donor (Scheme 2).^**[**20**]**^

### Co‐solvent

**Figure 1:**
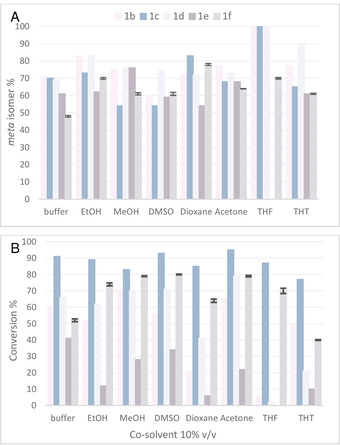
Methylation of 4‐subsituted catechols **1 b**–**f** in the absence and presence of 10 % v/v co‐solvent (EtOH, MeOH, DMSO, dioxane, acetone, THF, THT). **A)** Bars represent percentage of formed *m*‐**2 b**–**f** of the total amount of products **2 b**–**f. B)** Bars represent conversion. For reaction conditions see Scheme 2 (and experimental part). The error was between 0.6 and 1 % (average ±0.8 %).

Investigating organic solvents, the focus was set on water miscible solvents to ensure a homogeneous reaction system. Six water miscible organic solvents were tested such as ethanol, methanol, DMSO, acetone, 1,4‐dioxane and THF. 4‐Substituted catechols were chosen as substrate bearing initially hydrophilic groups (carboxylic acid, alcohol, aldehyde) as in substrates **1 b**–**1 f**. The solvents were tested at 10 % v/v (Figure 1).

The results in buffer only, thus in the absence of co‐solvent, are given for comparison, whereby in buffer the hydroxyl moiety in *meta*‐position to the substituent R was preferentially methylated giving isomer *m*‐**2 b**–**e** with 60–70 % in the product mixture (Figure 1). Substrate **1 f** bearing a primary alcohol moiety at an ethyl group led to a slight excess of the *para*‐product (52 %).

The two hydroxy functionalized solvents, ethanol and methanol, led to slightly improved regioselectivity in comparison to the results obtained in buffer, thus the relative amount of *m*‐**2 b**–**f** increased. Also, the conversions were comparable to the ones obtained in buffer except for **1 e**, which led to lower conversion (Figure 1B, Table S1).

DMSO is known to be well tolerated by biocatalysts^[21]^ and indeed the conversion in the presence of DMSO (10 % v/v) turned out to be similar to the values in the absence of co‐solvent. The regioselectivity was also comparable, thus DMSO did not have a benefit nor a disadvantage with respect to regioselectivity. The same was true for acetone leading to 64–77 % methylation on the phenolic OH in *meta* position to the substituent (Figure 1A); the overall conversion benefited slightly from the use of this organic co‐solvent. Switching to ethers such as 1,4‐dioxane and THF, more pronounced effects on the regioselectivity were found. An increased preference for the *meta*‐isomers *m*‐**2 c** and *m*‐**2 f** (83/17 and 78/22 *m/p* respectively) was observed with dioxane. Surprisingly, best regioselectivity was observed with THF for **1 b**–**d** leading to the formation of exclusively one regioisomer, namely *m*‐**2 b**–**d**. For substrate **1 c** even 87 % conversion was reached. For the other substrates, the increase in regioselectivity went in hand with reduced conversion.

Since THF was so beneficial with respect to regioselectivity, we tested also the related thio compound tetrahydrothiophene (THT), although it is barely water soluble leading to a two‐phase system; nevertheless, two substrates (**1 b** and **1 d**) benefited from the presence of THT as a two‐phase system leading e. g. to the formation of 90 % of *m*‐**2 d** (Figure 1A).

**Figure 2:**
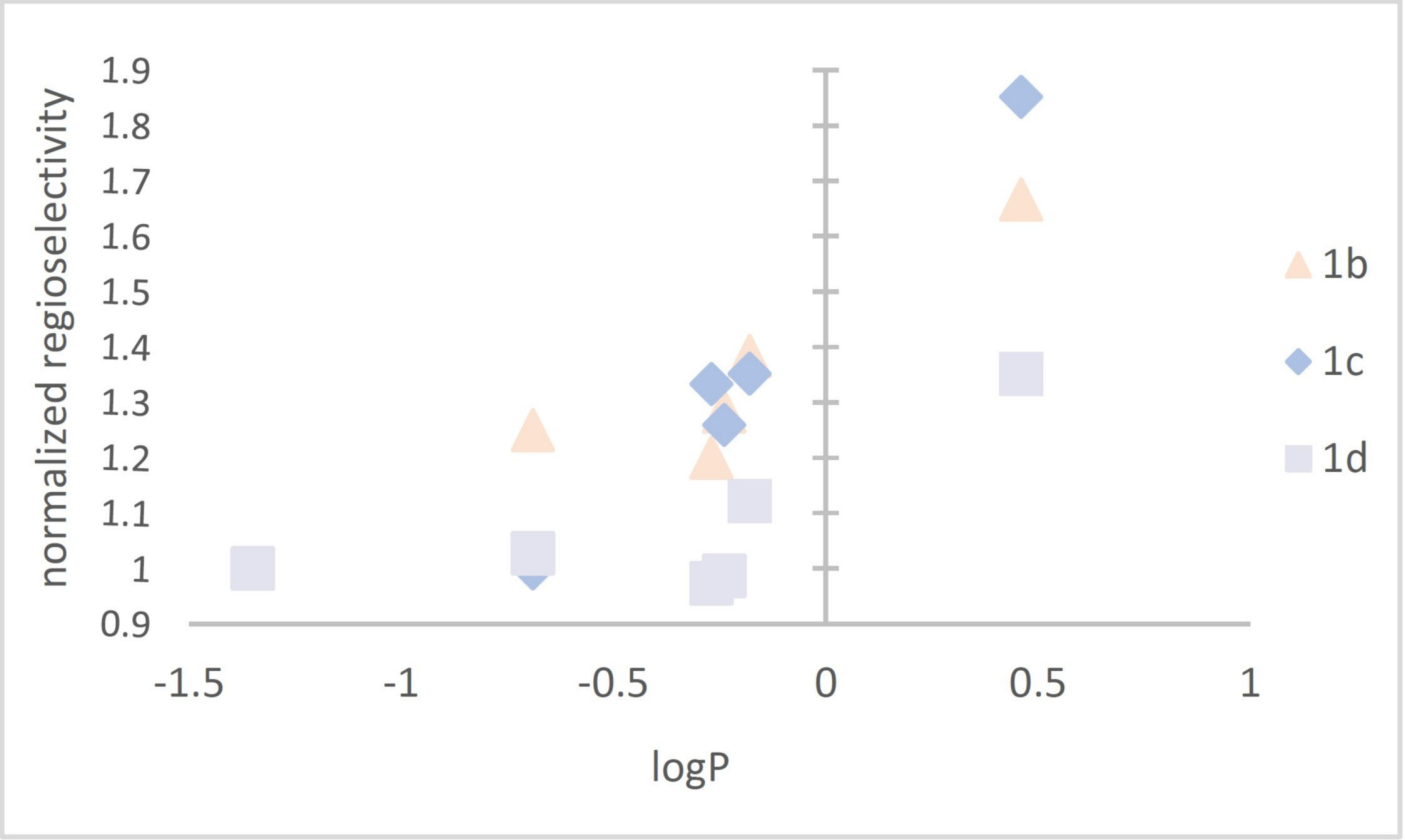
Normalized regioselectivity ( % *m‐* **2** formed over % *m‐* **2** formed in DMSO) plotted against LogP of water miscible co‐solvents used.

Plotting a normalized selectivity (percentage of *m*‐**2** formed over percentage *m*‐**2** formed in DMSO) for substrates **1 b**, **1 c** and **1 d** against logP, one can notice that mostly the increasing logP goes in hand with an increased selectivity (Figure 2).

The selectivity is highest in THF, which corresponds to a logP value of 0.46, whereas the most negative logP value (DMSO, logP: −1.35) was often corresponding to the lowest selectivity values (or one of the lowest).This trend is also observable for **1 g**, however, not so clear for the other substrates tested (**1 e**–**i**, Table S5).

Since THF as co‐solvent enabled a regioselective transformation at 10 % v/v for the substrates **1 b**, **1 c** and **1 d**, but with reduced conversion (especially for **1 b** and **1 d**), THF was subsequently tested at a lower concentration. At 5 % v/v, THF enabled an increase of conversion for both **1 b** and **1 d**, but the regioselectivity dropped (Table S2). The conversion of **1 c** was again high (90 %) however just 81 % of the product was the *meta*‐isomer *m*‐**2 c**. The co‐solvents THF, EtOH and MeOH were also investigated at higher concentration. Thereby THF did not lead to detectable product formation when used at 15 % v/v with the substrates **1 b** and **1 d**. Interestingly, still a 98/2 ratio of *m*/*p*‐**2 c** and 25 % conversion was observed for the transformation of **1 c**. When using 15 % v/v of EtOH, exclusively the isomer *m*‐**2 d** was observed at low conversion (14 %).

### Substrate

**Figure 3:**
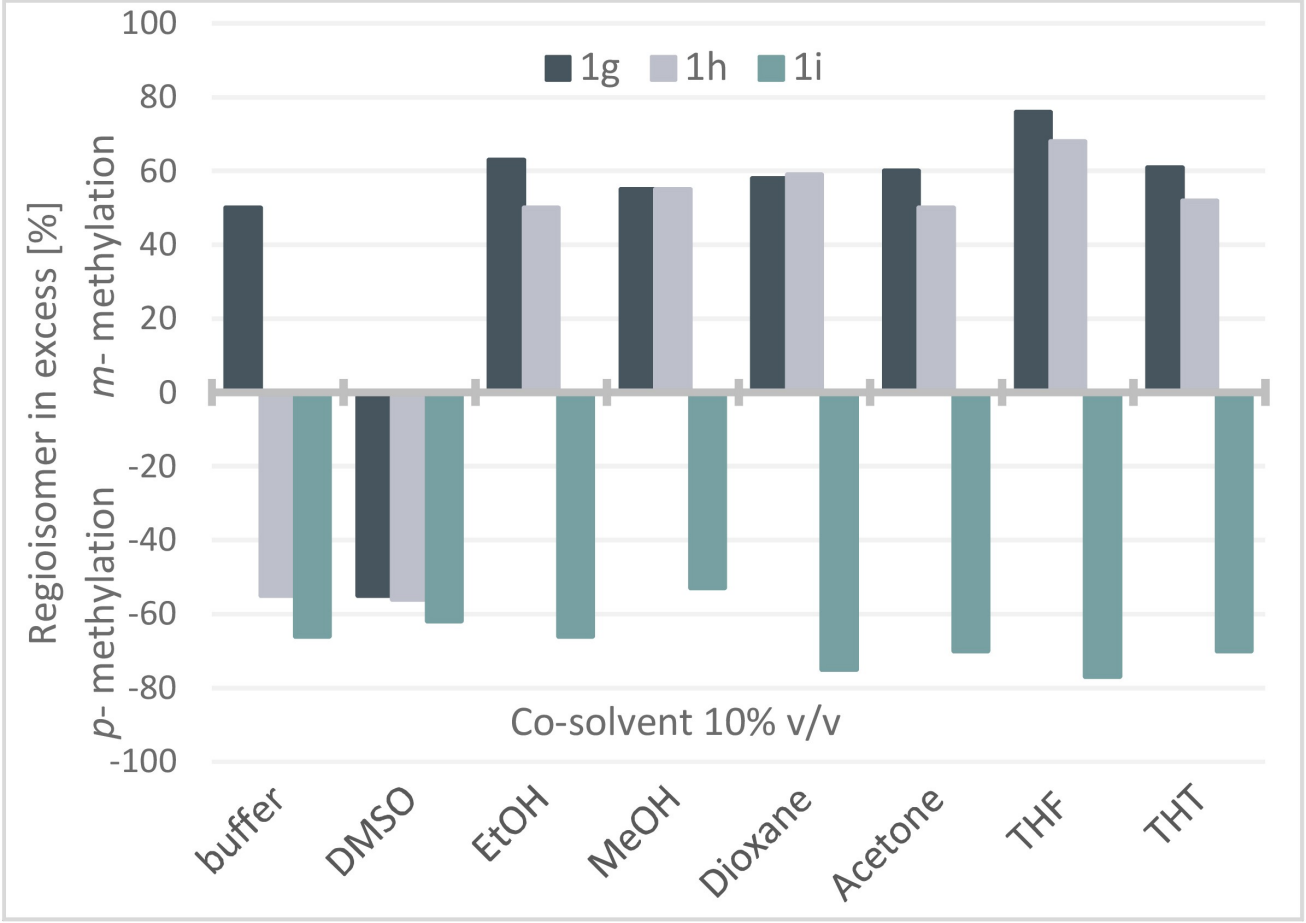
Methylation of substrates **1 g**–**i** in buffer and in the presence of 10 % v/v co‐solvents. The regioisomer formed in excess is given as percentage of the total amount of products **2 g**–**i** for the methylation of 4‐subsituted catechols **1 g**–**i**. For the reaction conditions see Scheme 2. The error was between 0.6 and 1 % (average ±0.8 %).

In the study above, substrates with a polar side chain R were investigated. The observed influence on regioselectivity,^[22]^ may be a result of the positioning of the substrate in the enzyme. To continue to investigate the influence of the side chain, three substrates with an apolar side chain were studied such as 4‐methylcatechol (**1 g**), 4‐ethylcatechol (**1 h**) and 4‐*tert*‐butylcatechol (**1 i**). It turned out that they were well accepted (conversions between 35 % and 70 %, Table S1). In the absence of co‐solvent, a mixture of ∼1 : 1 *m*/*p* was found for **1 g** (50.1 % of *m*‐**2 g**, Figure 3). In contrast to that and all previous results, substrate **1 h** bearing an ethyl group in 4‐position led to methylation of the OH located in *para*‐position to the substituent giving *p*‐**2 h** in slight excess (55 %). This effect was more pronounced when increasing the size of the substituent to a *tert*‐butyl group: substrate **1 i** led to 66 % of *para*‐methylation (product *p*‐**2 i**).

Testing the organic co‐solvents for these three substrates, it was found that the reversed regioselectivity for substrate **1 i** was preserved independently from the solvent investigated; **1 i** was methylated in *para*‐position with respect to the *tert*‐butyl group in the presence of all solvents tested and the ratio increased up to 77 % in the presence of 10 % v/v THF. Interestingly, all three substrates **1 g**–**i** were methylated preferentially in *para*‐position in DMSO, thus also substrate **1 g**, which led in general to *meta*‐methylation, was methylated in *para*‐position. In the presence of all other co‐solvents the two substrates with the small substituent (methyl, ethyl‐group **1 g**–**h**) were methylated at the *meta*‐position with respect to the substituent.

The conversions reached were in general comparable to the reaction without co‐solvent, except for THF and dioxane. As observed for other substrates (**1 b**–**f**), the use of THF led to reduced conversion; however, it has to be emphasized that the reduction of conversion went in hand with an increased regioselectivity for all three substrates. The percentage of *m*‐**2 g** and *m*‐**2 h** was improved as well as the regioselectivity for **1 i** leading preferentially to *p*‐**2 i**. This is in line with the results of the substrates bearing a polar side chain.

Although the effect of THF is of interest, one can only speculate on the molecular reason. The solvents affect the dielectric constant of the media, which may already induce subtle structural changes of the overall protein structure. Furthermore, the solvent molecules may coordinate to structural elements of the protein, thereby again leading to a change of structure; lastly, they may act like a decoy molecule in the active site, thus influencing the available space and influencing selectivity and reactivity.^[23]^

### Influence of the pH

**Figure 4:**
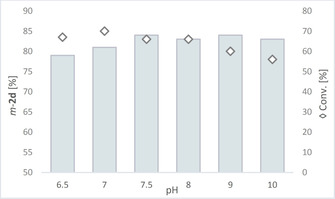
Regioisomer formed in excess (represented as bars) given as percentage of the total amount of products **2 d** for the methylation of **1 d** in MOPS buffer at varied pH values. The diamonds ◊ indicate conversion. For the reaction conditions see Scheme 2.

When looking at the pKa values for 4‐substituted catechols, there is a clear difference between the two phenolic OHs present in *para*‐ and *meta*‐position to the substituent.^[24]^ For instance, compound **1 d** is first deprotonated in *para*‐position [pKa(OH_p_)=8.71] and then in *meta*‐position [pKa(OH_m_)=11.78] according to literature.^[24]^ Therefore, in the case the methylation mechanism would involve ionic species and deprotonation of the phenol to enable a nucleophilic attack at the methyl bound to the cobalamin and if no steric control by the enzyme is involved, one might expect methylation preferentially in *para* position. Nevertheless, as observed above, the biocatalytic methylation of **1 d** showed regioselectivity towards the *meta*‐position. Consequently, the pH of the reaction mixture was varied in order to study its influence on the regioselectivity. Substrate **1 d** and **1 i** were employed as model substrates to have one member of the substrates with a polar side chain and one with an apolar one. Interestingly, an enhancement of regioselectivity towards the *meta*‐isomer was observed when going from pH 6.5 to pH 9 for **1 d** (Figure 4).

Increasing the pH above pH 7 led to slightly lower conversion, whereby at pH 10 still 56 % conversion was observed.

**Figure 5:**
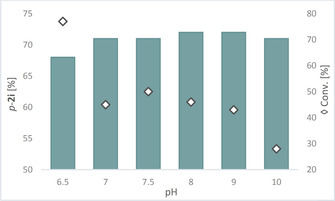
Regioisomer formed in excess (represented as bars) given as percentage of the total amount of products **2 i** for the methylation of **1 i** in MOPS buffer at varied pH values. The diamonds ◊ indicate conversion. For the reaction conditions see Scheme 2.

The order of pKa values for compound **1 i** is reverse to the one of **1 d**, thus for this substrate the *meta*‐position [pKa(OH_m_)=8.24] is first deprotonated and then the *para*‐position [pKa(OH_p_)=13.51].^[24]^ Nevertheless, the enzyme directs again here to the less favored position, namely to the *para*‐position. Increasing the pH from pH 6.5 to e. g. pH 8 leads to comparable values the relative amount of *p*‐**2 i** (68 to 72, Figure 5). Thus, the pH plays not a dominant role in selectivity and deprotonation orders seemed to be overruled by the orientation of the substrate in the active site, which can, at the current stage, not be explained in better detail since no crystal structure is available yet.

Finally, the effect of pH and co‐solvents were tested in combination for substrate **1 d**. At pH 7.5 and 9 the bio‐methylation was performed in the presence of ethanol (10 % v/v). Indeed, almost solely vanillin was observed (92 % and 94 % respectively), indicating that these reaction parameters may have additive effects (Table S3).

## Conclusions

The regioselectivity of the biocatalytic cobalamin‐dependent methylation was studied by testing the influence of polar and apolar substituents at position 4 of catechol, the influence of water miscible co‐solvents and the pH. Regioselectivity and conversion improved by the use of several co‐solvents, depending on the concentration added (e. g. EtOH and MeOH). It is worth to note, that, in the presence of THF (10 % v/v) exclusively a single regioisomer was observed in selected cases leading to the methylation of the OH in *meta*‐position only. Furthermore, substrates bearing an apolar and bulky side chain in position 4 of catechol underwent *O*‐methylation in *para* position, which opposite to the substrates bearing a polar side chain, which were methylated preferentially in *meta*‐position.

## Experimental Section

### General Information

^1^H and ^13^C NMR spectra were recorded at 20 °C on a 300 MHz Bruker NMR. The conversions and regiomeric ratio of all biotransformations were measured by reversed‐phase HPLC with an Agilent 1260 Infinity HPLC system at 25 °C, using a Phenomenex Luna® 5 mm, C18, 100 A (250×4.6 mm) column. Detection was performed with a diode array detector (G4212B). TLC was carried out with pre‐coated aluminum sheets (TLC Silica gel 60 F254, Merck) with detection by UV (254 nm) and/or by staining with cerium molybdate solution. All chemicals and solvents were obtained from commercial suppliers (TCI, Sigma Aldrich/Fluka, VWR International/Merck, Roth) and used as received unless stated otherwise. Commercial available compounds were utilized as obtained as references for the quantitative and qualitative determination of the isomers produced during the biotransformation. In the case that the references were not commercially available, they were synthesized. 3‐Hydroxytyrosol (**1 f**) was synthesized following a procedure from literature.^[25]^ Units are defined as converted substrate in μmol per min.

### Synthetic Procedures

**3,4‐Dihydroxyphenyl)acetic methyl ester 5 f**. Concentrated H_2_SO_4_ (5 drops) was added to a solution of 3,4‐dihydroxyphenylacetic acid (1 g, 5.95 mmol) in MeOH (95 mL) under argon atmosphere in the dark and the reaction was refluxed for 2 h. After solvent evaporation under reduced pressure, the residue was redissolved in EtOAc and washed with sat. aqu. NaHCO_3_ (20 ml). The aqueous phase was extracted with EtOAc (3×30 mL), and the combined organic extracts were washed with brine (30 ml), dried over Na_2_SO_4_, and evaporated under reduced pressure to give the pure ester **5 f** (900 mg, 5.3 mmol, yield 84 %). ^1^H NMR (300, MeOD) δ 6.70–6.68 (m, 2H), 6.58–6.52 (m, 1H), 3.66 (s, 3H), 3.46 (s, 2H). ^13^C NMR (75 MHz, MeOD) δ 174.53, 146.27, 145.42, 126.89, 121.60, 117.30, 116.26, 52.36, 41.15.

**2‐(2,2‐Dimethylbenzo[1,3]dioxol‐5‐yl)acetic methyl ester 4 f**. Under argon atmosphere in the dark, 2,2‐dimethoxypropane (6.5 mL, 53 mmol, 9 eq) and *p*‐toluene sulfonic acid (186 mg, 0.98 mmol, 0.16 eq) were added to a solution of **5 f** (900 mg, 5.3 mmol, 1 eq) in anhydrous CH_2_Cl_2_ (180 mL), and the solution was refluxed for 8 h. The reaction mixture was neutralized by shaking with sat. aqu. NaHCO_3_ (30 ml), and the resulting aqueous phase was extracted three times with CHCl_3_ (3×20 mL). The combined organic extracts were dried (Na_2_SO_4_) and evaporated under reduced pressure. The crude residue was purified via column chromatography using silica gel (20 : 1, w/w) by elution with cyclohexane/EtOAc (8 : 2 v/v) to furnish pure **4 f** (849 mg, 3.82 mmol, yield 72 %). ^1^H NMR (300 MHz, MeOD) δ 6.65 (d, *J*=5.3 Hz, 3H), 3.66 (s, 3H), 3.52 (s, 2H), 1.63 (s, 6H). ^13^C NMR (75 MHz, MeOD) δ 174.17, 148.91, 147.89, 128.51, 122.90, 119.02, 110.40, 108.86, 52.42, 41.32, 25.91.

**2‐(2,2‐Dimethylbenzo[1,3]dioxol‐5‐yl)ethanol 3 f**. A solution of ester 4 f (849 mg, 3.82 mmol) in anhydrous Et_2_O (16 mL) was added dropwise to a solution of dry Et_2_O (20 mL) and LiAlH_4_ (77.4 mg, 2.05 mmol) under Ar atm at 0 °C. The suspension was left stirring for 3 h at 0 °C. The mixture was then cooled, and excess hydride was cautiously decomposed by adding an aqueous NaOH solution (0.75 M, 350 μL), until the formation of a white precipitate occurred; then the solution was dried by adding Na_2_SO_4_. After removal of the precipitate and Na_2_SO_4_ by filtration under reduced pressure over a celite pad, the solution was evaporated under reduced pressure to obtain a crude mixture. The material was purified over silica gel (20 : 1, w/w) by elution with cyclohexane/ethyl acetate (7 : 3, v/v) to give pure **3 f** (299 mg, 1.65 mmol, yield 43 %). ^1^H NMR (300 MHz, CDCl_3_) δ 6.69–6.56 (m, 3H), 3.79 (t, 2H), 2.75 (t, 2H), 1.64 (s, 6H). ^13^C NMR (75 MHz, CDCl_3_) δ 147.83, 146.25, 131.55, 121.42, 117.90, 109.25, 108.29, 63.95, 39.07, 26.02.

**Hydroxytyrosol 1 f**. Amberlyst 15 (350 mg) was added to a solution of 3 f (299 mg, 1.65 mmol) in MeOH (23 mL), and the suspension was refluxed under stirring for 8 h while the reaction progress was monitored by TLC. Finally, the resin was removed by filtration, and the resulting solution was evaporated under reduced pressure to the crude product which was purified via column chromatography cyclohexane/EtOAc (8 : 2 v/v) to afford **1 f** (201 mg, 1.3 mmol, yield 78 %). Spectroscopic data were superimposable with those of the pure standard of **1 f**. ^1^H NMR (300 MHz, MeOD) δ 6.66 (m, *J*=7.3, 5.0 Hz, 1H), 6.52 (m, *J*=8.0, 2.1 Hz, 2H), 3.67 (t, *J*=7.3 Hz, 2H), 2.66 (t, *J*=7.2 Hz, 2H). ^13^C NMR (75 MHz, MeOD) δ 146.14, 144.62, 131.77, 121.19, 117.05, 116.29, 64.60, 39.67.

**5‐(2‐hydroxyethyl)‐2‐methoxyphenol** ***p*** **‐2 f**. A solution of commercially available 2‐(3‐hydroxy‐4‐methoxyphenyl)acetic acid (315 mg, 1.5 mmol) in anhydrous THF (5 mL) was added dropwise over 15 min to a solution of dry THF (5 mL) and LiAlH_4_ (236 mg, 6 mmol) under Ar atm and 0 °C. The suspension was left stirring for 4 h at 0 °C. The mixture was then cooled, and excess hydride was cautiously decomposed by adding NaOH (0.75 M, 1 mL), until the formation of a white precipitate occurred; then the solution was dried (Na_2_SO_4_). After removal of the precipitate and Na_2_SO_4_ by filtration under reduced pressure over a celite pad, and washed with MeOH the solution was evaporated under reduced pressure to obtain a crude mixture. The material was purified over silica gel (20 : 1, w/w) by elution with cyclohexane/EtOAc (7 : 3 v/v) to give pure *p*‐**2 f** (103 mg, 0.61 mmol, yield 41 %). ^1^H NMR (300 MHz, MeOD) δ 6.90–6.81 (m, 1H), 6.76–6.63 (m, 2H), 3.84 (s, 3H), 3.73 (t, *J*=7.2 Hz, 2H), 2.73 (t, *J*=7.2 Hz, 2H). ^13^C NMR (75 MHz, MeOD) δ 147.50, 147.40, 133.19, 121.09, 116.99, 112.84, 64.46, 56.47, 39.63.

**5‐(1‐hydroxyethyl)‐2‐methoxyphenol** ***p*** **‐3 h**. Isovanillin (6.6 mmol, 1 g, 1 eq) previously dissolved in anh. THF (4 ml) was added dropwise over 15 minutes to a stirred solution of MeMgI (3 M in Et_2_O, 13.2 mmol, 4.4 mL, 2 eq) under Ar atm at 0 °C. The mixture was left stirring for 3 hours at 35 °C. It was then cooled to 0 °C again and aqueous HCl (1 M, 6.6 mL) was carefully added. The resulting aqueous phase was extracted with EtOAc (3×35 mL). The combined organic fractions were dried using Na_2_SO_4_. After removal of Na_2_SO_4_ by filtration, the solution was evaporated under reduced pressure. After purification via column chromatography (c‐hex:EtOAc, 7 : 3 v/v) *p*‐**3 h** was obtained as a yellow oil (2.7 mmol, 410 mg, 31 % yield). ^1^H NMR (300 MHz, MeOD) δ 6.85 (dd, *J*=5.3, 2.9 Hz, 2H), 6.78 (dd, *J*=8.3, 2.0 Hz, 1H), 4.70 (q, *J*=6.5 Hz, 1H), 3.82 (s, 3H), 1.39 (d, *J*=6.5 Hz, 3H). ^13^C NMR (75 MHz, MeOD) δ 148.10, 147.36, 140.49, 117.81, 113.67, 112.46, 70.53, 56.43, 25.46.

**5‐ethyl‐2‐methoxyphenol** ***p*** **‐2 h**. Pd/C (10 % wt, 0.44 mmol, 462 mg, 0.16 eq) was added to a stirred solution of *p‐*3 h (2.7 mmol, 410 mg, 1 eq) in MeOH (90 ml), then HClO_4_ (70 % wt, 895 μl) was added. The mixture was left stirring for 16 hours at room temperature under H_2_ atmosphere (1 atm). The crude mixture was filtrated over celite and then NaHCO_3_ (sat. aqu. solution, 75 ml) was added. The resulting aqueous phase was extracted with CH_2_Cl_2_ (3×40 ml), dried over Na_2_SO_4_ and evaporated under reduced pressure. The crude product was then purified via column chromatography (*c*‐hexane/EtOAc, 7 : 3 v/v) to obtain the pure *p*‐**2 h** as brown oil (1.44 mmol, 219 mg, 59 % yield). ^1^H NMR (300 MHz, MeOD) δ 6.77 (d, *J*=8.1 Hz, 1H), 6.69–6.52 (m, 2H), 3.78 (s, 3H), 2.48 (q, *J*=7.6 Hz, 2H), 1.15 (t, *J*=7.6 Hz, 3H).^13^C NMR (75 MHz, MeOD) δ 147.32, 147.00, 138.55, 119.78, 115.89, 112.85, 56.49, 29.19, 16.36.

### Enzyme Expression

Expression of required proteins was performed with *E. coli* using the plasmids pEG457 (MTase I) and pEG459 (CP) previously reported.^[16a]^ Over‐night cultures (ONCs) were prepared in 50 mL falcon tubes by supplementing LB‐medium (20 mL) with ampicillin (100 μg/ml). After inoculation with a single colony of transformed cells the cultures were incubated overnight at 37 °C and 120 rpm. Main cultures (500 mL volume in 2 L non‐baffled Erlenmeyer flask) supplemented with ampicillin (100 μg/ml) were inoculated with the appropriate volume of ONC to an initial OD_600_ of 0.05. After incubation at 37 °C and 120 rpm (2 hours) until an OD_600_ of 0.6–0.8 was reached, expression of the target protein was induced by the addition of AHTC (0.2 μg/mL). Shaking was continued at 120 rpm and the temperature of 25 °C overnight. After shaking for 24 h at the optimal temperature, the cells were harvested by centrifugation (4000 rpm, 4 °C, 20 min). The cell pellets were re‐suspended in 50 mM TRIS‐HCl buffer pH 7 yielding a 15 wt % 15 wt% cell suspension. Next, the cells were disrupted by sonication (Branson Digital Sonifier®) using the following settings: duration 2 min, 2.0 s sonicate, 1.0 s pause, 40 % amplitude. While sonication was performed (at least 3 times with 30 seconds breaks in between), the cells were constantly cooled with ice. Afterwards by centrifugation for 15 minutes at 14500 rpm, the cell fragments were removed from the extract. The cell‐free extract was finally lyophilized and stored at 4 °C, ready to be used for biotransformations.

### Biotransformations Using CFE

Preparation of *holo*‐CP. The functional *holo*‐CP was obtained by reconstitution of the corrinoid protein with exogenous cofactor (methyl cobalamin) under inert atmosphere as already described.^[16b]^ For this purpose, methyl cobalamin (2 mM) was dissolved in the presence of betaine (3 M) and DTT (2 mM) in TRIS/HCl buffer (50 mM, pH 7, 0.5 mM DTT, 0.1 mM PMSF) to form a reconstitution buffer. Then, freeze‐dried CP (100 mg/mL CFE) was loaded with the freshly prepared reconstitution buffer (1 mL) and incubated for at least 2 h at 4 °C to allow incorporation of the cofactor. Afterwards salts and unbound cobalamin were removed using a PD MidiTrap^TM^ G‐25 column (GE Healthcare) according to the manual provided by the manufacturer. Finally, reconstituted *holo*‐CP was eluted with MOPS/KOH buffer (100 mM, pH 6.5, 150 mM KCl) yielding a red colored protein solution which was stored at 4 °C until further use.

Biocatalytic methylation. Biocatalytic reactions were performed in degassed buffers under inert atmosphere (N_2_ 5.0) in a MBraun LABstar glove box equipped with a MB‐OX‐EC O_2_‐sensor. Analytical biotransformations were carried out on 120 μL scale as follows: freeze‐dried MTase I (32–80 mU, 40 mg/ml CFE) was rehydrated in *holo*‐CP solution (400 μL/mL, 66 mg/ml CFE). Donor 2a (final concentration 50 mM) and acceptors 1b‐i (final concentration 10 mM) dissolved in MOPS/KOH buffer (50 mM, pH 6.5, containing 150 mM KCl) were finally added. Reaction samples were shaken at 30 °C and 800 rpm for 24 hours in an orbital shaker. The procedure was adapted depending on the parameters investigated (e. g. pH, co‐solvent addition).

Semi‐preparative scale biotransformation. Biocatalytic reactions were performed in degassed buffers under inert atmosphere (N_2_ 5.0) in a MBraun LABstar glove box equipped with a MB‐OX‐EC O_2_‐sensor. Semi‐preparative scale biotransformations were carried out on 24 mL scale as follows: freeze‐dried MTase I (32–80 mU, 40 mg/ml CFE) was rehydrated in *holo*‐CP solution (400 μL/mL, 66 mg/ml CFE). Donor 2a (final concentration 50 mM) and acceptors 1b‐i (final concentration 10 mM) dissolved in MOPS/KOH buffer (50 mM, pH 6.5, containing 150 mM KCl) were finally added. Reaction samples were shaken at 30 °C and 800 rpm for 24 hours. The crude mixture was divided in four falcon tubes (50 mL volume) and extracted with EtOAc (5 mL×3) while centrifuging (4000 rpm, 20 minutes, room temperature). The combined organic fractions were combined and dried over Na_2_SO_4_. The solvent was finally evaporated under reduced pressure.

Determination of conversions and regioisomeric ratio. After reaction, an aliquot (90 μL) from each sample was quenched by adding acetonitrile (MeCN, 540 μL). After incubation (15 min) at room temperature H_2_O (HPLC grade, 270 μL) was added and the denatured protein was removed by centrifugation (14.000 rpm, 15 min). The supernatant was filtered over a cotton pad and analyzed by HLPC (Agilent 1260 Infinity system equipped with an UV detector) using a C18 column (Phenomenex, Luna, C18 100c, 250×4.6 mm, 5 mm). H_2_O/MeCN (+0.1 % TFA) was used as eluent with a flow rate of 1 mL/min. Compounds were detected by UV‐absorption and conversions were calculated based on calibrations curves (for example of calibration curves see Supporting Information). Identification of the isomers was performed by spiking commercial or synthesized reference materials with products from biotransformation (example reported in Supporting Information). The mixture were eluted with H_2_O/MeCN (+0.1 % TFA) gradient with a flow rate of 1 mL/min with one of this methods: method A. 100 % H_2_O (hold 2 min), gradient from 0–40 % MeCN (13 min), gradient to 100 % MeCN (5 min, hold 2 min), 100 % H_2_O (hold 3 min); method B. 100 % H_2_O (hold 2 min), gradient from 0–30 % MeCN (13 min), gradient to 100 % MeCN (5 min, hold 2 min), 100 % H_2_O (hold 3 min); method C. 100 % H_2_O (hold 2 min), gradient from 0–70 % MeCN (13 min), gradient to 100 % MeCN (5 min, hold 2 min), 100 % H_2_O (hold 3 min); method D. 100 % H_2_O (hold 2 min), gradient from 0–60 % MeCN (33 min), gradient to 100 % MeCN (5 min, hold 2 min), 100 % H_2_O (hold 3 min).

## Conflict of interest

The authors declare no conflict of interest.

## Supporting information

As a service to our authors and readers, this journal provides supporting information supplied by the authors. Such materials are peer reviewed and may be re‐organized for online delivery, but are not copy‐edited or typeset. Technical support issues arising from supporting information (other than missing files) should be addressed to the authors.

Supplementary
