# Supplementary material for: Regioselectivity of Cobalamin‐Dependent Methyltransferase Can Be Tuned by Reaction Conditions and Substrate
Source: ChemCatChem. 2020 Oct 1;12(23):5977–83. doi: 10.1002/cctc.202001296 (PMC7783988; doi:10.1002/cctc.202001296)
Supplement: Supplementary file 1 — Supplementary [file CCTC-12-5977-s001.pdf]

ChemCatChem

Supporting Information

Regioselectivity of Cobalamin-Dependent Methyltransferase Can Be Tuned by Reaction Conditions and Substrate

Simona Pompei, Christopher Grimm, Judith E. Farnberger, Lukas Schober, and Wolfgang Kroutil*

Table of Content

Figure S1. 3

Table S1. 4

Table S2. 4

Figure S2. 5

Table S3. 5

Table S4. 5

Table S5. 6

Scheme S1. Synthesis of reference and starting material (for procedures see main paper) 7

NMRs 8

Figure S3. (3,4-Dihydroxyphenyl)acetic methyl ester **5f** ^{1}H NMR. 8

Figure S4. 3,4-Dihydroxyphenyl)acetic methyl ester **5f** ^{13}C NMR. 9

Figure S5. 2-(2,2-Dimethylbenzo[1,3]dioxol-5-yl)acetic methyl ester **4f** ^{1}H NMR. 10

Figure S6. 2-(2,2-Dimethylbenzo[1,3]dioxol-5-yl)acetic methyl ester **4f** ^{13}C NMR. 11

Figure S7. 2-(2,2-Dimethylbenzo[1,3]dioxol-5-yl)ethanol **3f** ^{1}H NMR. 12

Figure S8. 2-(2,2-Dimethylbenzo[1,3]dioxol-5-yl)ethanol **3f** ^{13}C NMR. 13

Figure S9. Hydroxytyrosol **1f** ^{1}H NMR. 14

15

Figure S10. Hydroxytyrosol **1f** ^{13}C NMR. 15

Figure S11. 5-(2-hydroxyethyl)-2-methoxyphenol *p*-**2f** ^{1}H NMR. 16

Figure S12. 5-(2-hydroxyethyl)-2-methoxyphenol *p*-**2f** ^{13}C NMR. 17

Figure S13. 5-(1-hydroxyethyl)-2-methoxyphenol *p*-**3h** ^{1}H NMR. 18

19

Figure S14. 5-(1-hydroxyethyl)-2-methoxyphenol *p*-**3h** ^{13}C NMR. 19

Figure S15. 5-ethyl-2-methoxyphenol *p*-**2h** ^{1}H NMR. 20

Figure S16. 5-ethyl-2-methoxyphenol *p*-**2h** ^{13}C NMR. 21

Figure S17. Example of HPLC chromatograms. 22

Figure S18. Example of HPLC chromatograms of biotransformations. 23

Figure S19. Example of HPLC chromatograms of biotransformations with **XXXYX**. 25

Figure S20. (**A**) HPLC chromatogram of biotransformation and (**B**) spiked chromatogram with the commercial available reference isomer 4-(tert-butyl)-2-methoxyphenol. 26

Figure S21. 26

Figure S22. Example of HPLC calibration curve. 27

Table S4. HPLC retention times and used methods for the screened substrates and products. 28

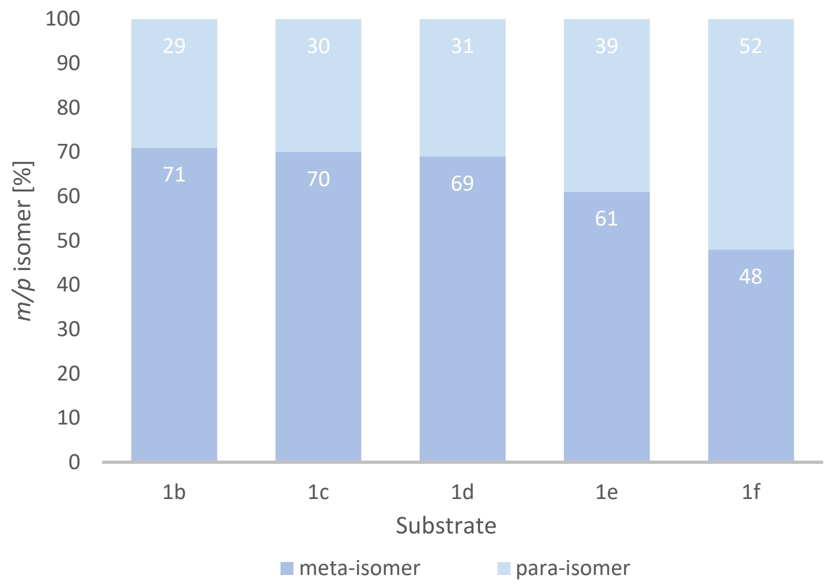

Figure S1. Regioselectivity in buffer for substrates **1b-f**.

	Buffer		DMSO		MeOH		THF		Dioxane		EtOH		Acetone		THT	
**Substr**	conv.[a] [%]	**2n** [b] [%]	conv [%]	**2n** [%]	conv [%]	**2n** [%]	conv [%]	**2n** [%]	conv [%]	**2n** [%]	conv [%]	**2n** [%]	conv [%]	**2n** [%]	conv [%]	**2n** [%]
**1b**	60	71	60	60	70	75	6	100	21	72	52	83	65	77	50	77
**1c**	91	70	93	54	83	54	87	100	85	83	89	73	95	68	77	65
**1d**	66	69	71	74	70	76	2	100	41	72	62	83	79	73	21	90
**1e**	41	61	34	59	28	76	n.d.	n.d.	6	54	12	62	22	68	10	61
**1f**	52	48	80	61	79	61	70	70	64	78	74	70	79	64	40	61
**1g**	71	50	68	-55	79	55	2	76	27	58	58	63	70	60	75	61
**1h***	75	-55	81	-56	75	55	9	68	19	59	39	50	68	50	78	52
**1i***	38	-66	83	-62	75	-53	2	-77	11	-75	12	-66	22	-70	43	-70

Table S1. Co-solvent screening (10% v/v). [a] Conversion (conv.%) of acceptor into products after 24 hours based on the limiting reagent. *Conversion based on catechol formation. [b] The regioisomer in excess (indicated as **2n**) was calculated after 24 hours as the percentage of the one isomer out of the total of products formed (considered as 100%). Positive values indicate the *m*-**2n** to be in access, whereas negative values denote *p*-**2n** to be in access. The error was between 0.6 and 1% (average ±0.8%).

	THF 5% v/v conv.[a] [%]	*m*-**2**[b] [%]	THF 15% v/v conv.[%]	*m*-**2**[%]	EtOH 15% v/v conv.%	*m*-**2**%	MeOH 15% v/v conv.%	*m*-**2**%
**1b**	30	73	n.d.	n.d.	20	68	-	-
**1c**	90	81	25	98	-	-	-	-
**1d**	68	76	n.d.	n.d.	14	100	-	-
**1e**	n.d.	n.d.	n.d.	n.d.	-	-	21	6000

Table S2. Co-solvent screening (5-15% v/v). [a] Conversion (conv. [%]) of acceptor into products after 24 hours based on the limiting reagent. [b] The regioisomer in excess (*m*-**2**) was calculated after 24 hours as the percentage of the one isomer in the total of products formed (considered as 100%). The error was between 0.6 and 1% (average ±0.8%).

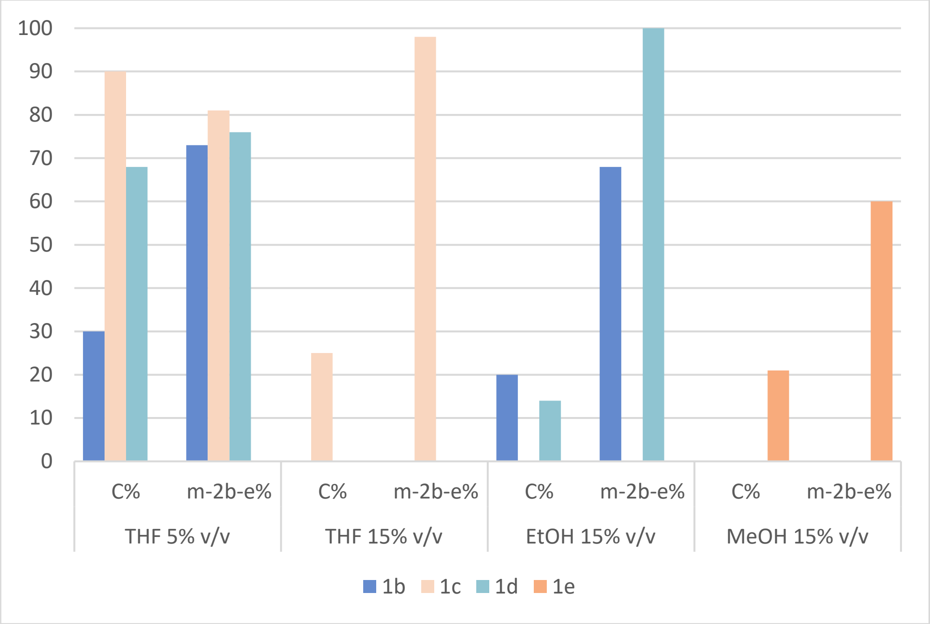

Figure S2. Regioselectivity for substrates **1b-e** with different co-solvents concentrations (5-15% v/v).

pH	*m*-**2d** [*b*][%]	conv. [a] [%]
7.5	92	8
9	94	6

Table S3. Additivity of pH and co-solvent (EtOH) effect. [a] Conversion (conv. [%]) of acceptor **1d** into products (*m*-**2d** and *p*-**2d**) after 24 hours based on the limiting reagent. [b] Vanillin (indicated as *m*-**2d**) was calculated after 24 hours as the percentage of the one isomer out of the total of products formed (considered as 100%).

	**1d**		**1i**	
pH	conv.[a] [%]	*m*-**2d**[b] [%]	conv.[%]	*p*-**2i** [%]
6.5	67	79	77	68
7.0	70	81	45	71
7.5	66	84	50	71
8.0	66	83	46	72
9.0	60	84	43	72
10.0	56	83	28	71

Table S4. Effect of the pH. [a] Conversion (conv. [%]) of acceptor **1d** into products (*m*-**2d** and *p*-**2d**) after 24 hours based on the limiting reagent. [b] Isomer (indicated as *m*-**2d** or *p*-**2i**) was calculated after 24 hours as the percentage of the one isomer out of the total of products formed (considered as 100%). The error was between 0.6 and 1% (average ±0.8%).

Co-solvent	logP	*m*-**2b** [%]	*m*-**2b** - normalized	*m*-**2c** [%]	*m*-**2c** - normalized	*m*-**2d** [%]	*m*-**2d** - normalized
DMSO	-1.35	60	1	54	1	74	1
methanol	-0.69	75	1.25	54	1	76	1.027027
dioxane	-0.27	72	1.2	72	1.333333	72	0.972973
acetone	-0.24	77	1.283333	68	1.259259	73	0.986486
ethanol	-0.18	83	1.383333	73	1.351852	83	1.121622
THF	0.46	100	1.666667	100	1.851852	100	1.351351

Co-solvent	logP	*m*-**2e** [%]	*m*-**2f** -[%]	*m*-*p*-**2g** [%]	*m*-*p*-**2h** [%]	*p*-**2i** [%]
DMSO	-1.35	59	59	-55	-56	-62
methanol	-0.69	76	61	55	55	-53
dioxane	-0.27	54	78	58	59	-75
acetone	-0.24	68	64	60	50	-70
ethanol	-0.18	62	70	63	50	-66
THF	0.46	-	70	76	68	-77

Table S5. LogP of water miscible co-solvents used and corresponding regioselectivity and normalized regioselectivity (% *m*-**2** formed over % *m*-**2** in DMSO). Positive values indicate the *m*-**2n**, whereas negative values denote *p*-**2n**. The error was between 0.6 and 1% (average ±0.8%).

For Hydroxytyrosol **1f:** (3,4-Dihydroxyphenyl)acetic methyl ester **5f**.

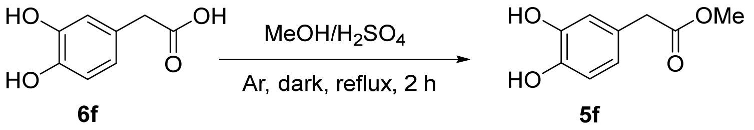

2-(2,2-Dimethylbenzo[1,3]dioxol-5-yl)acetic methyl ester **4f**.

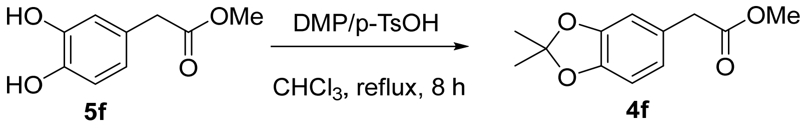

2-(2,2-Dimethylbenzo[1,3]dioxol-5-yl)ethanol **2f**.

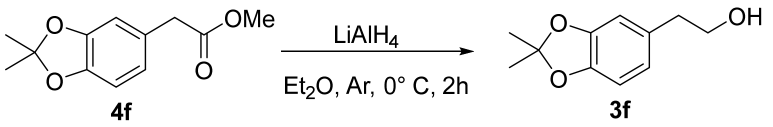

Hydroxytyrosol **1f**.[1]

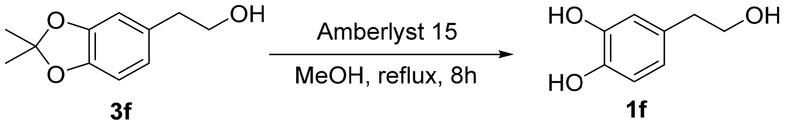

Reference material: 5-(2-hydroxyethyl)-2-methoxyphenol *p*-**2f**.

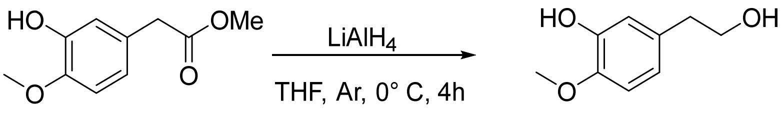

p-**2f**

Reference material: 5-(1-hydroxyethyl)-2-methoxyphenol *p*-**3h**.

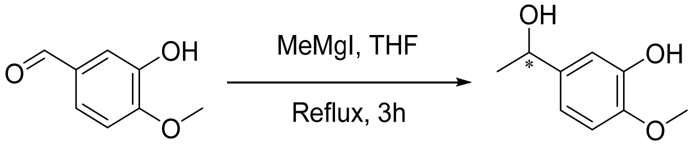

p-**2d** *p*-**3h**

5-ethyl-2-methoxyphenol *p*-**2h**.

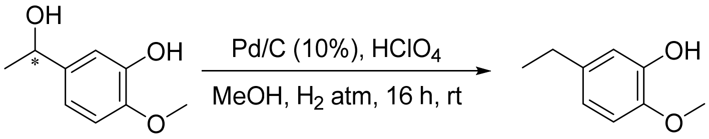

p-**3h** *p*-**2h**

Scheme S1. Synthesis of reference and starting material (for procedures see main paper)

NMRs

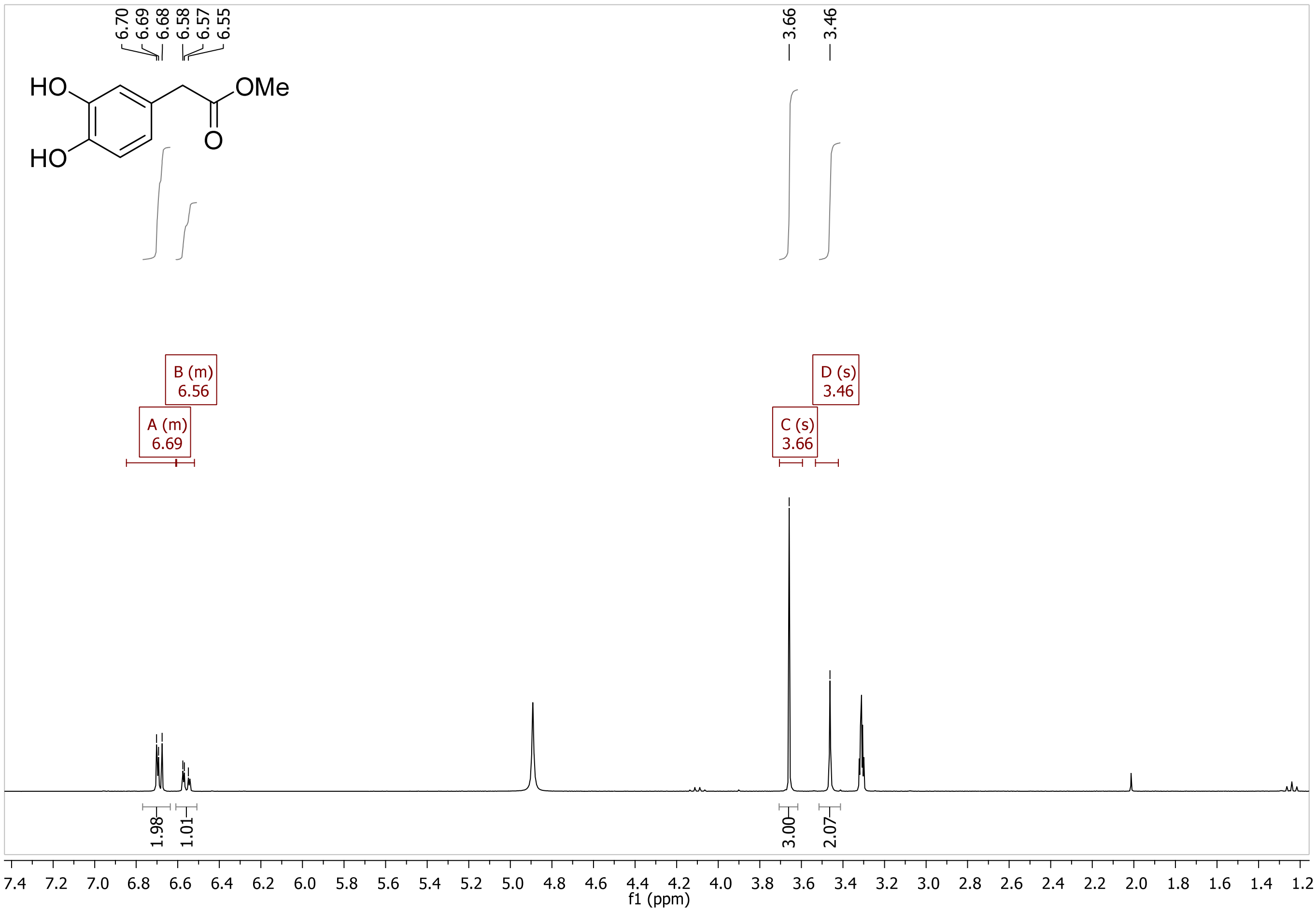

Figure S3. (3,4-Dihydroxyphenyl)acetic methyl ester **5f** ^{1}H NMR.

^{1}H NMR (300 MHz, MeOD) δ 6.70 – 6.68 (m, 2H), 6.58 – 6.52 (m, 1H), 3.66 (s, 3H), 3.46 (s, 2H).

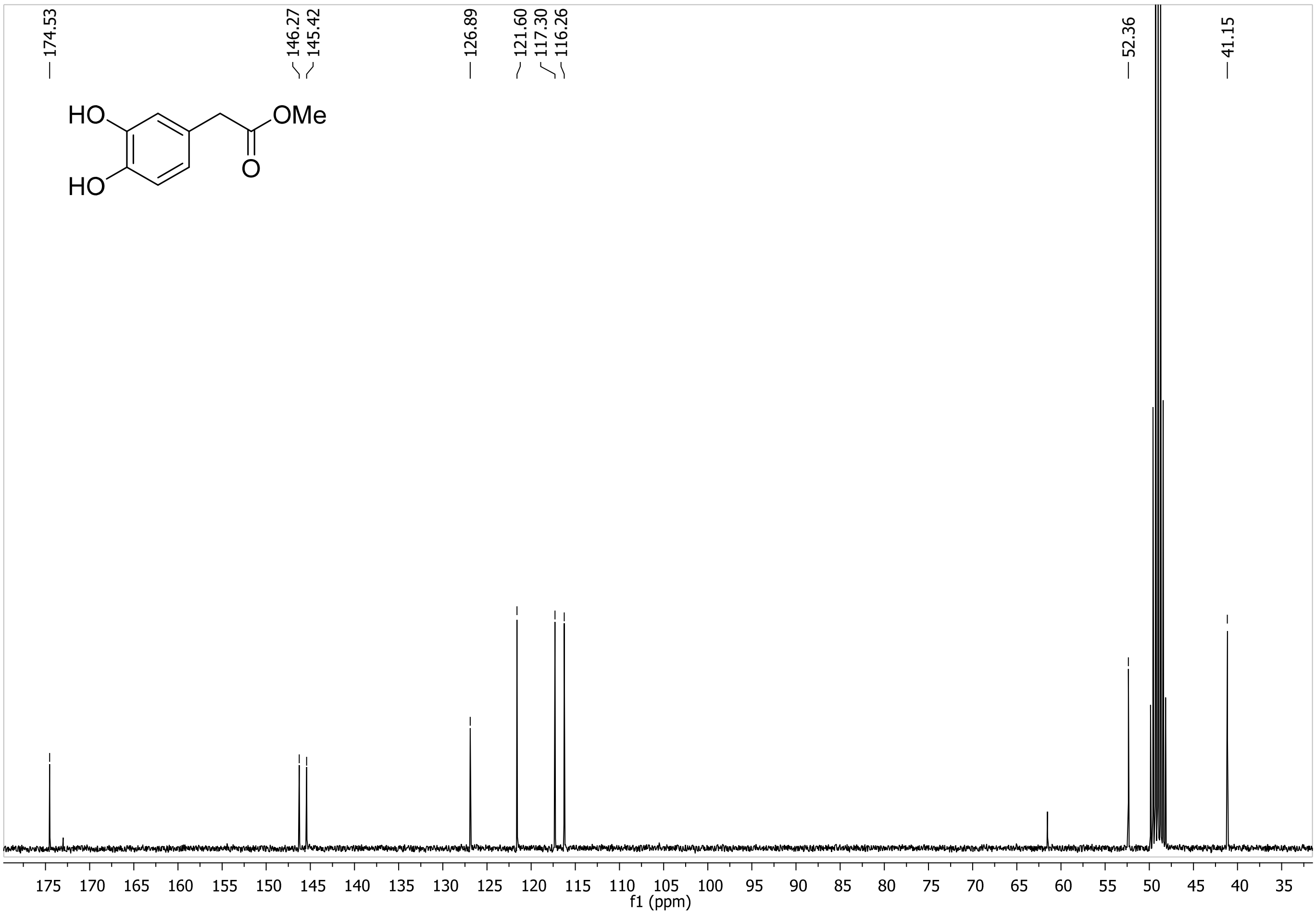

Figure S4. 3,4-Dihydroxyphenyl)acetic methyl ester **5f** ^{13}C NMR.
^{13}C NMR (75 MHz, MeOD) δ 174.53, 146.27, 145.42, 126.89, 121.60, 117.30, 116.26, 52.36, 41.15.

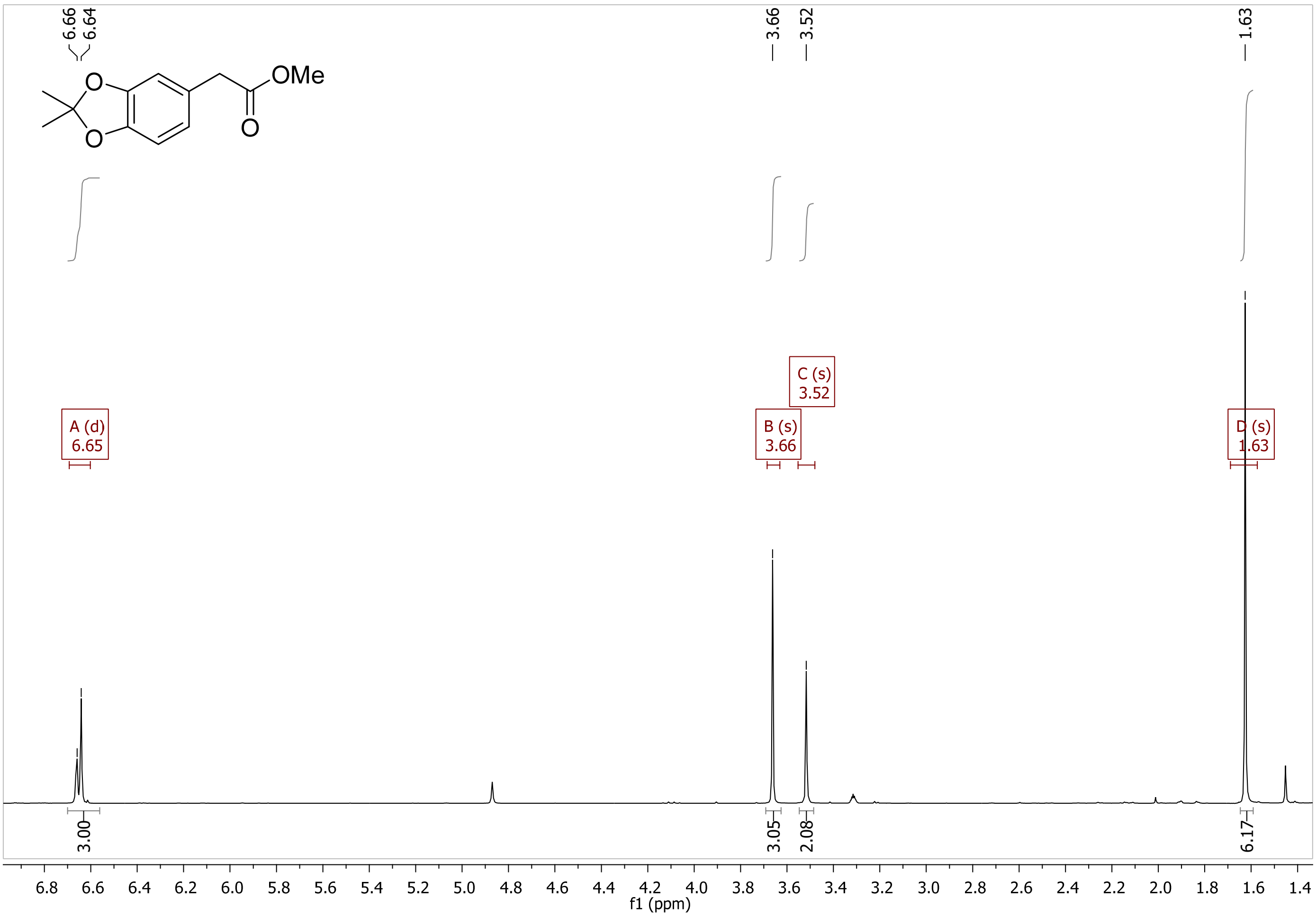

Figure S5. 2-(2,2-Dimethylbenzo[1,3]dioxol-5-yl)acetic methyl ester **4f** ^{1}H NMR.

^{1}H NMR (300 MHz, MeOD) δ 6.65 (d, *J* = 5.3 Hz, 3H), 3.66 (s, 3H), 3.52 (s, 2H), 1.63 (s, 6H).

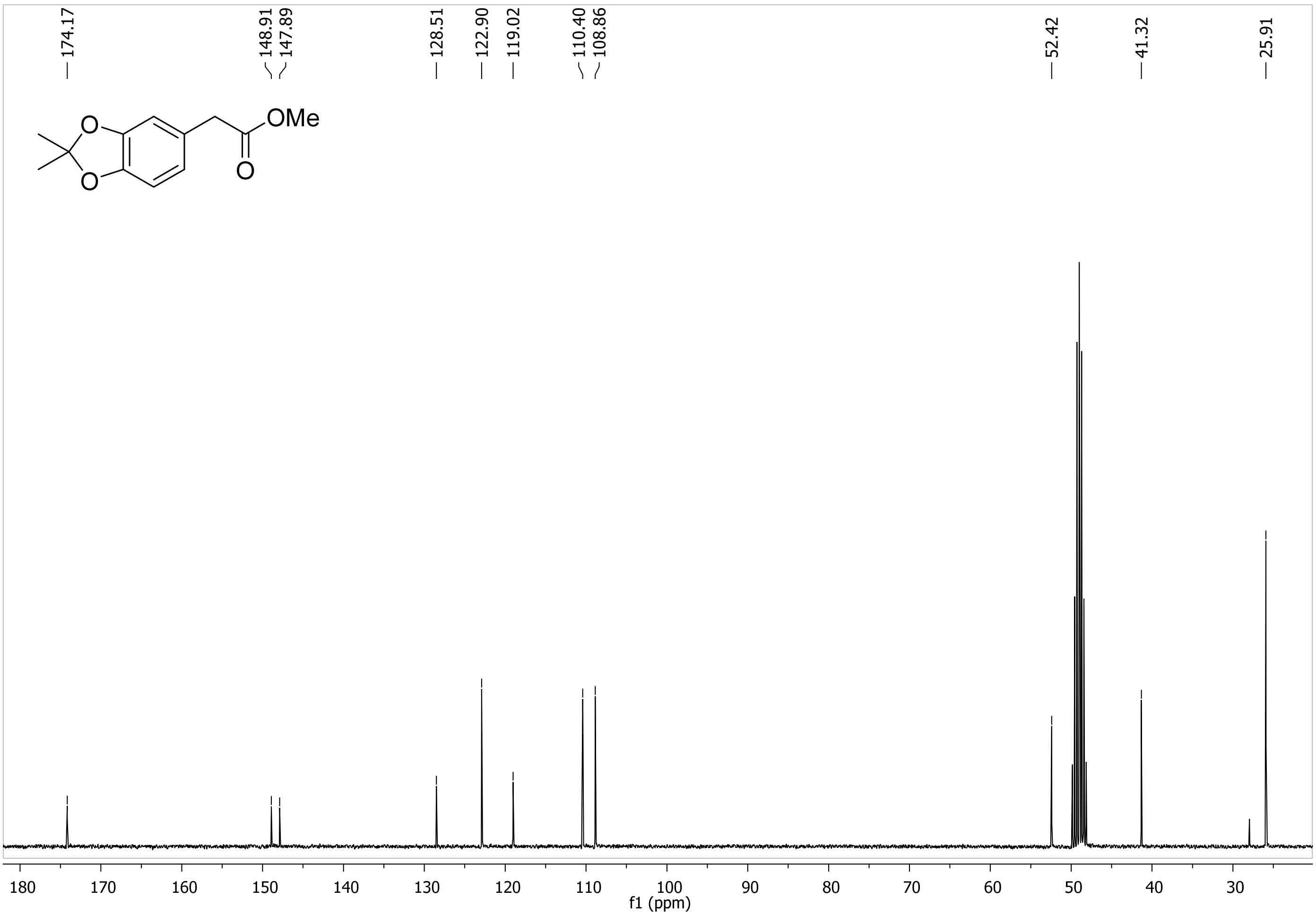

Figure S6. 2-(2,2-Dimethylbenzo[1,3]dioxol-5-yl)acetic methyl ester **4f** ^{13}C NMR.
^{13}C NMR (75 MHz, MeOD) δ 174.17, 148.91, 147.89, 128.51, 122.90, 119.02, 110.40, 108.86, 52.42, 41.32, 25.91.

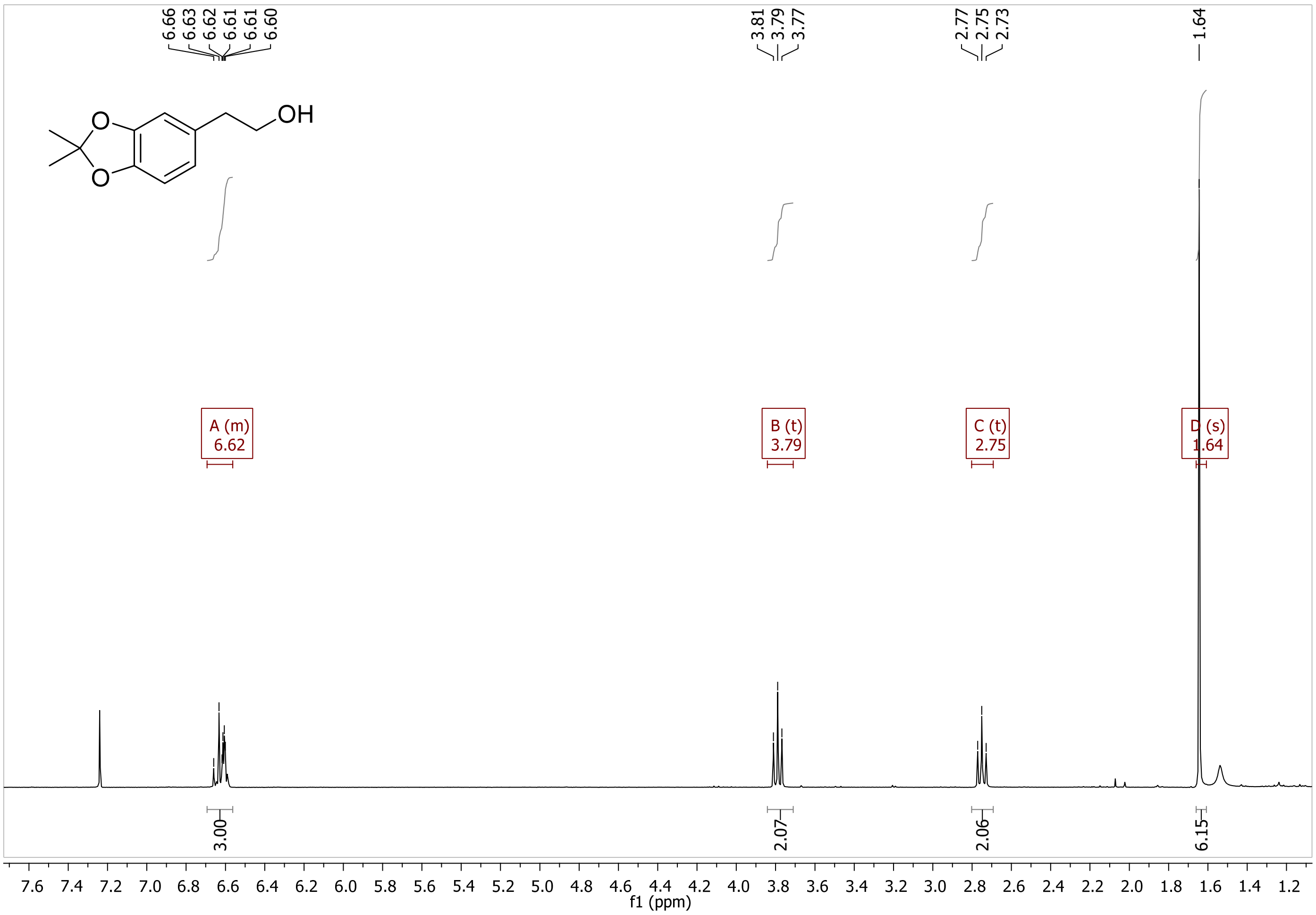

Figure S7. 2-(2,2-Dimethylbenzo[1,3]dioxol-5-yl)ethanol **3f** ^{1}H NMR.
^{1}H NMR (300 MHz, $CDCl_3$) δ 6.69 – 6.56 (m, 3H), 3.79 (t, 2H), 2.75 (t, 2H), 1.64 (s, 6H).

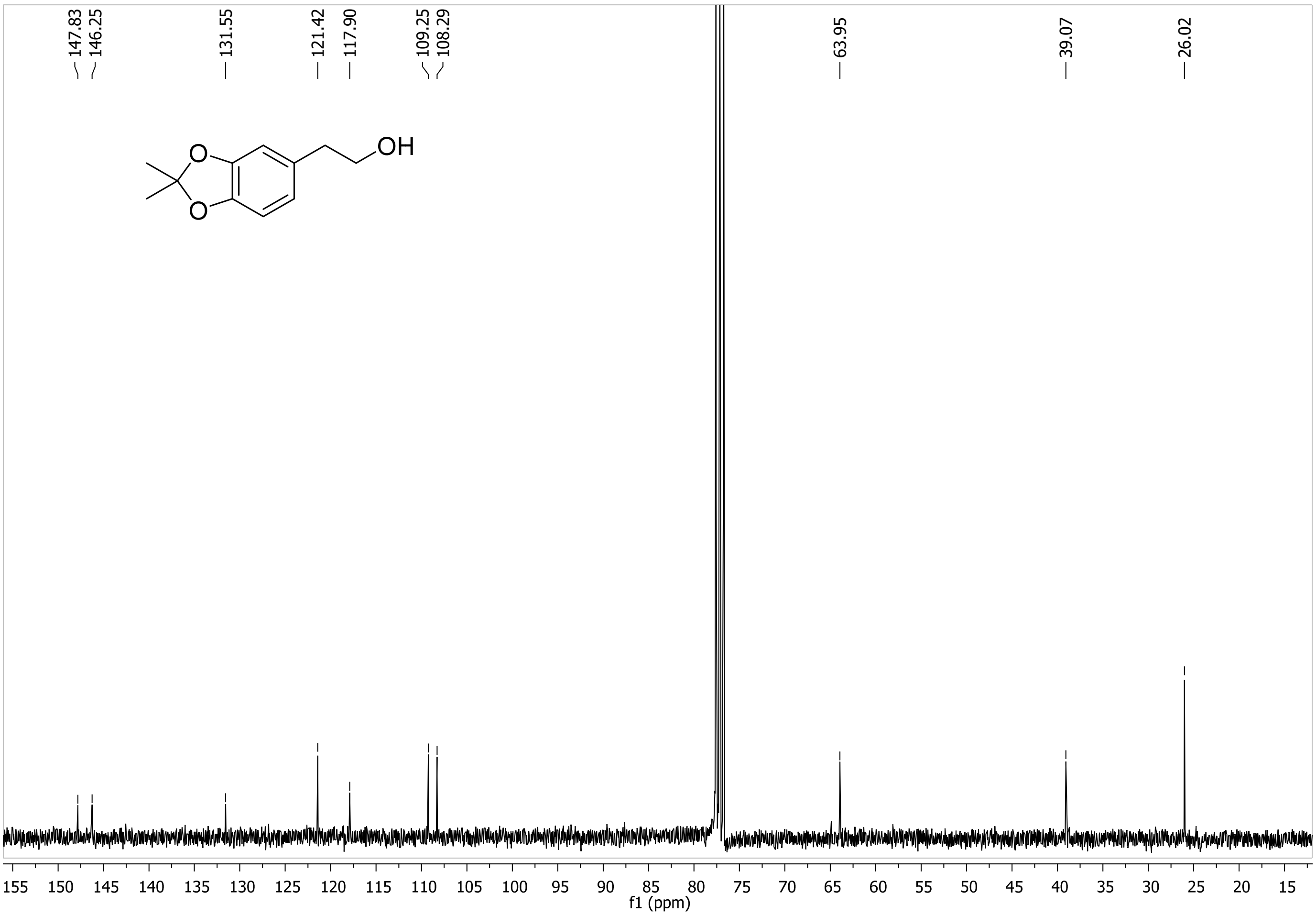

Figure S8. 2-(2,2-Dimethylbenzo[1,3]dioxol-5-yl)ethanol **3f** ^{13}C NMR.

^{13}C NMR (75 MHz, $CDCl_3$) δ 147.83, 146.25, 131.55, 121.42, 117.90, 109.25, 108.29, 63.95, 39.07, 26.02.

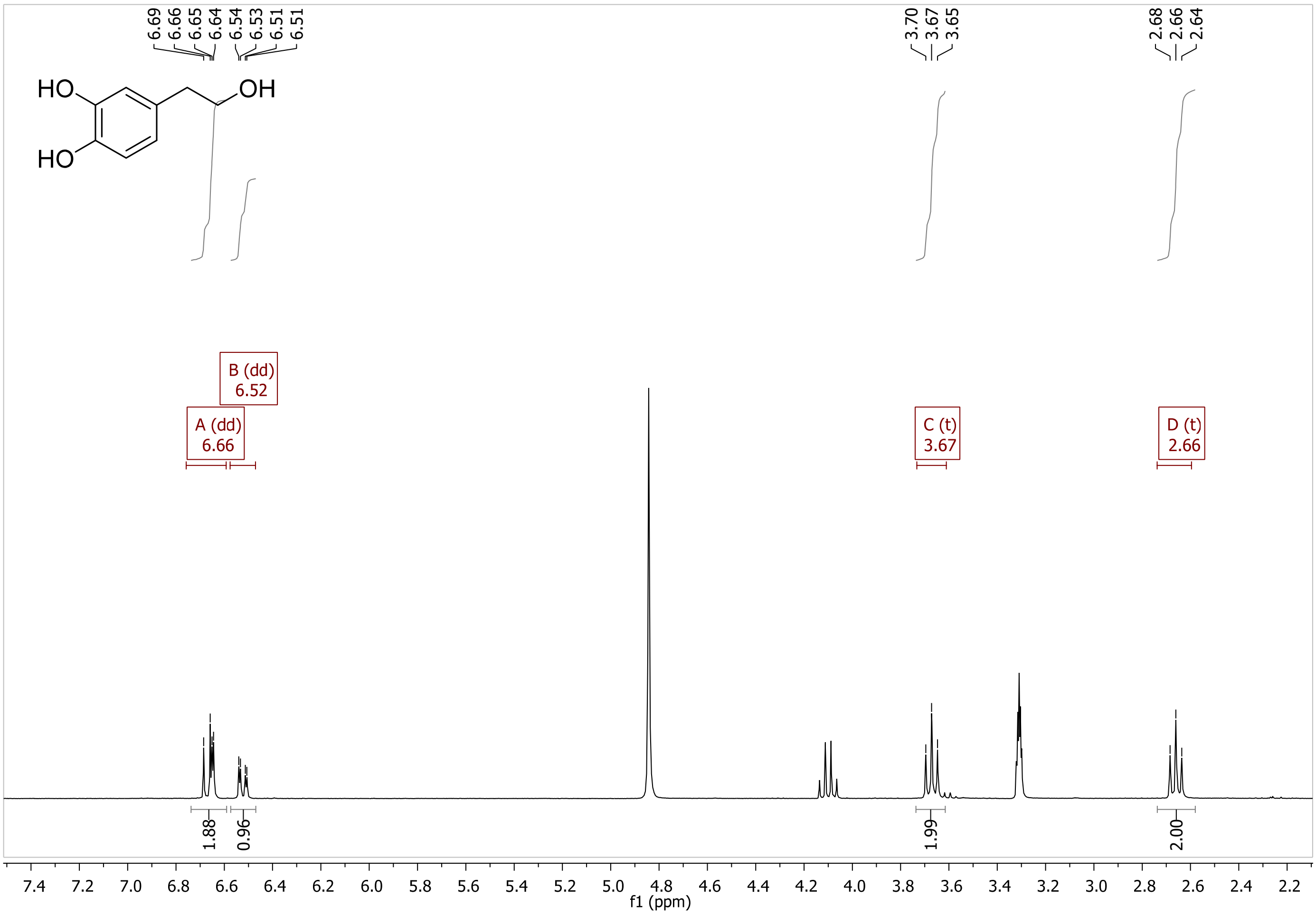

Figure S9. Hydroxytyrosol **1f** ^{1}H NMR.

^{1}H NMR (300 MHz, MeOD) δ 6.66 (m, *J* = 7.3, 5.0 Hz, 1H), 6.52 (m, *J* = 8.0, 2.1 Hz, 2H), 3.67 (t, *J* = 7.3 Hz, 2H), 2.66 (t, *J* = 7.2 Hz, 2H).

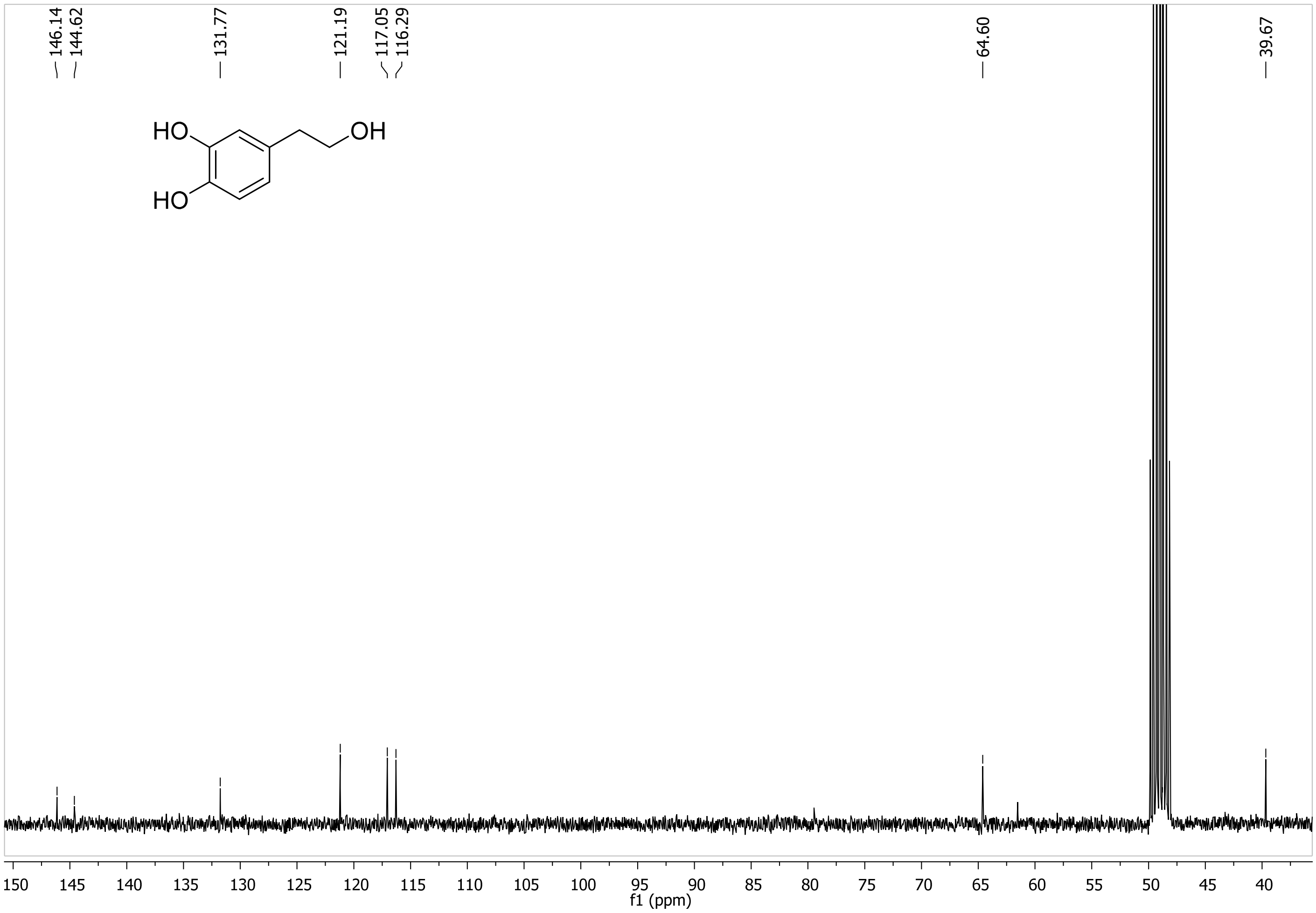

Figure S10. Hydroxytyrosol **1f** ^{13}C NMR.

^{13}C NMR (75 MHz, MeOD) δ 146.14, 144.62, 131.77, 121.19, 117.05, 116.29, 64.60, 39.67.

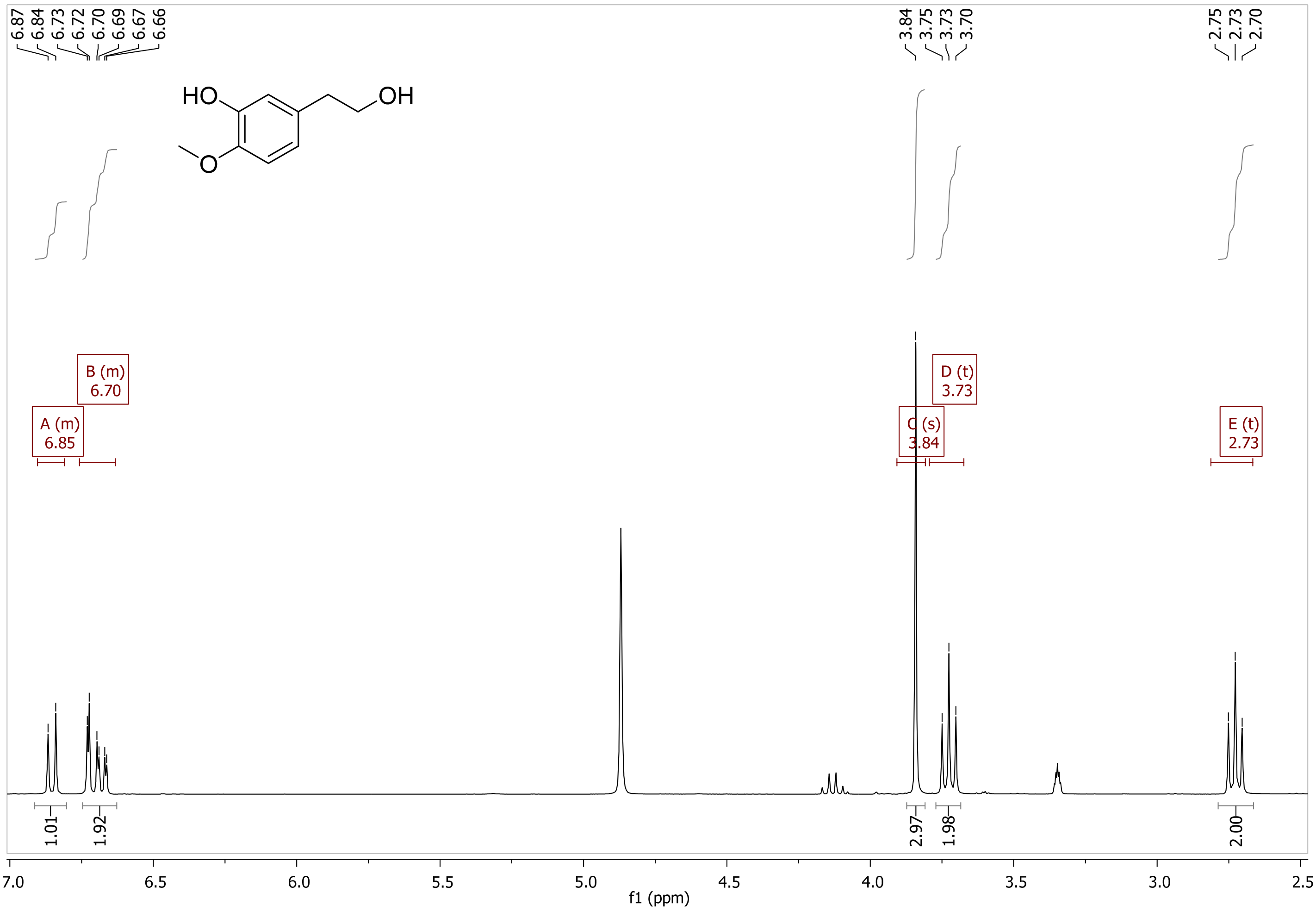

Figure S11. 5-(2-hydroxyethyl)-2-methoxyphenol *p*-**2f** ^{1}H NMR.
^{1}H NMR (300 MHz, MeOD) δ 6.90 – 6.81 (m, 1H), 6.76 – 6.63 (m, 2H), 3.84 (s, 3H), 3.73 (t, *J* = 7.2 Hz, 2H), 2.73 (t, *J* = 7.2 Hz, 2H).

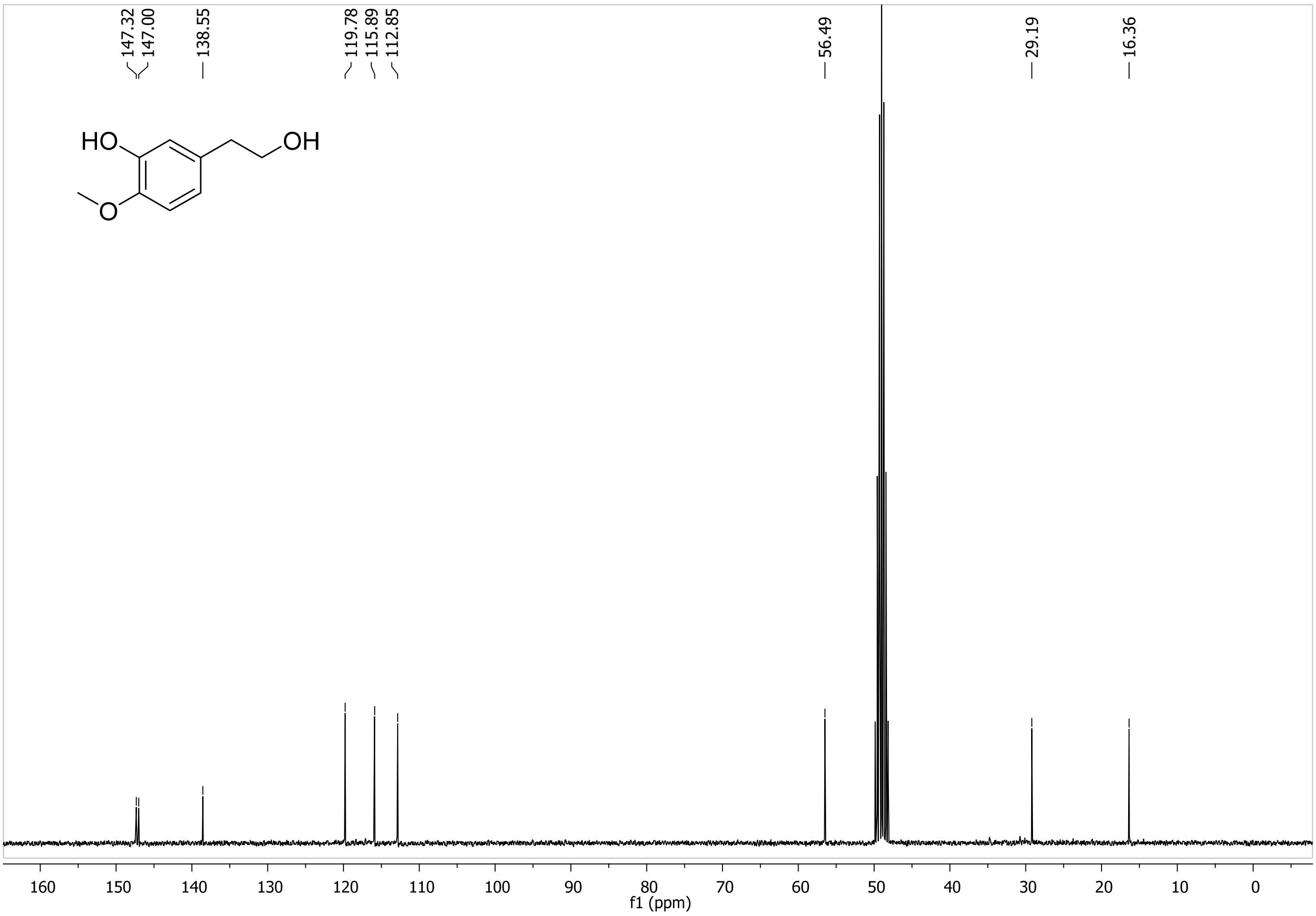

Figure S12. 5-(2-hydroxyethyl)-2-methoxyphenol *p*-**2f** ^{13}C NMR.
^{13}C NMR (75 MHz, MeOD) δ 147.50, 147.40, 133.19, 121.09, 116.99, 112.84, 64.46, 56.47, 39.63.

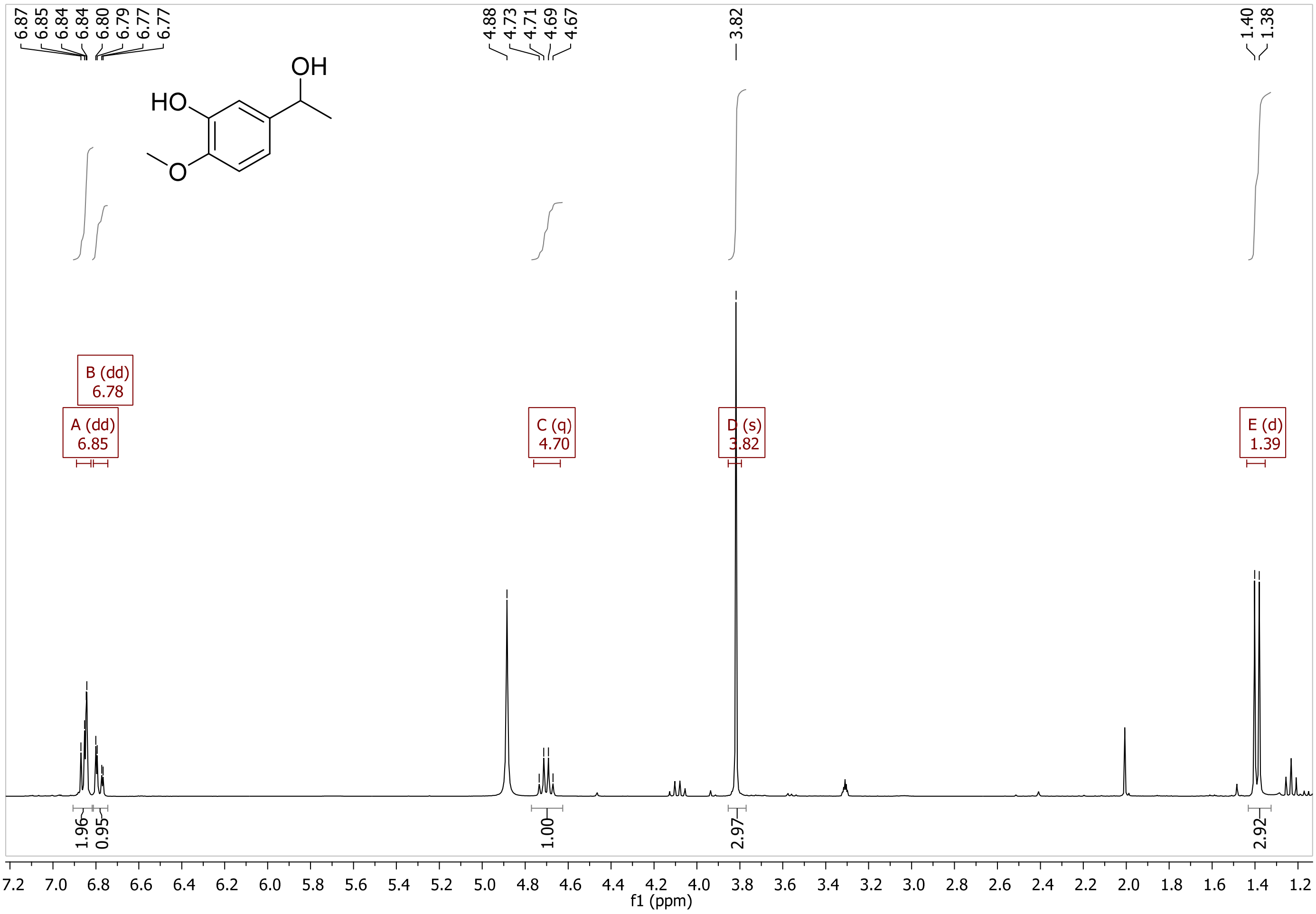

Figure S13. 5-(1-hydroxyethyl)-2-methoxyphenol *p*-**3h** ^{1}H NMR.
^{1}H NMR (300 MHz, MeOD) δ 6.85 (dd, J = 5.3, 2.9 Hz, 2H), 6.78 (dd, J = 8.3, 2.0 Hz, 1H), 4.70 (q, J = 6.5 Hz, 1H), 3.82 (s, 3H), 1.39 (d, J = 6.5 Hz, 3H).

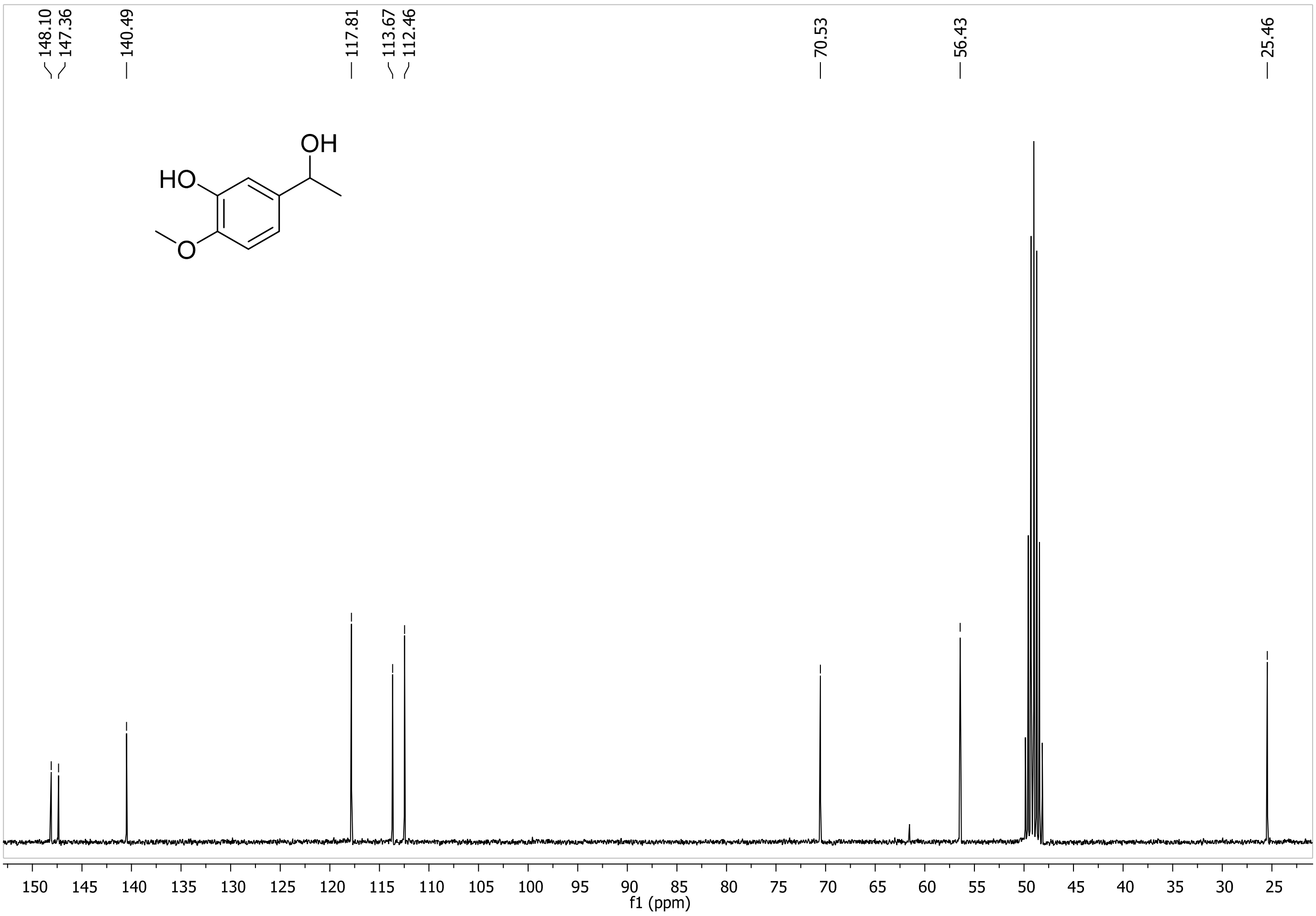

Figure S14. 5-(1-hydroxyethyl)-2-methoxyphenol *p*-**3h** ^{13}C NMR.

^{13}C NMR (75 MHz, MeOD) δ 148.10, 147.36, 140.49, 117.81, 113.67, 112.46, 70.53, 56.43, 25.46.

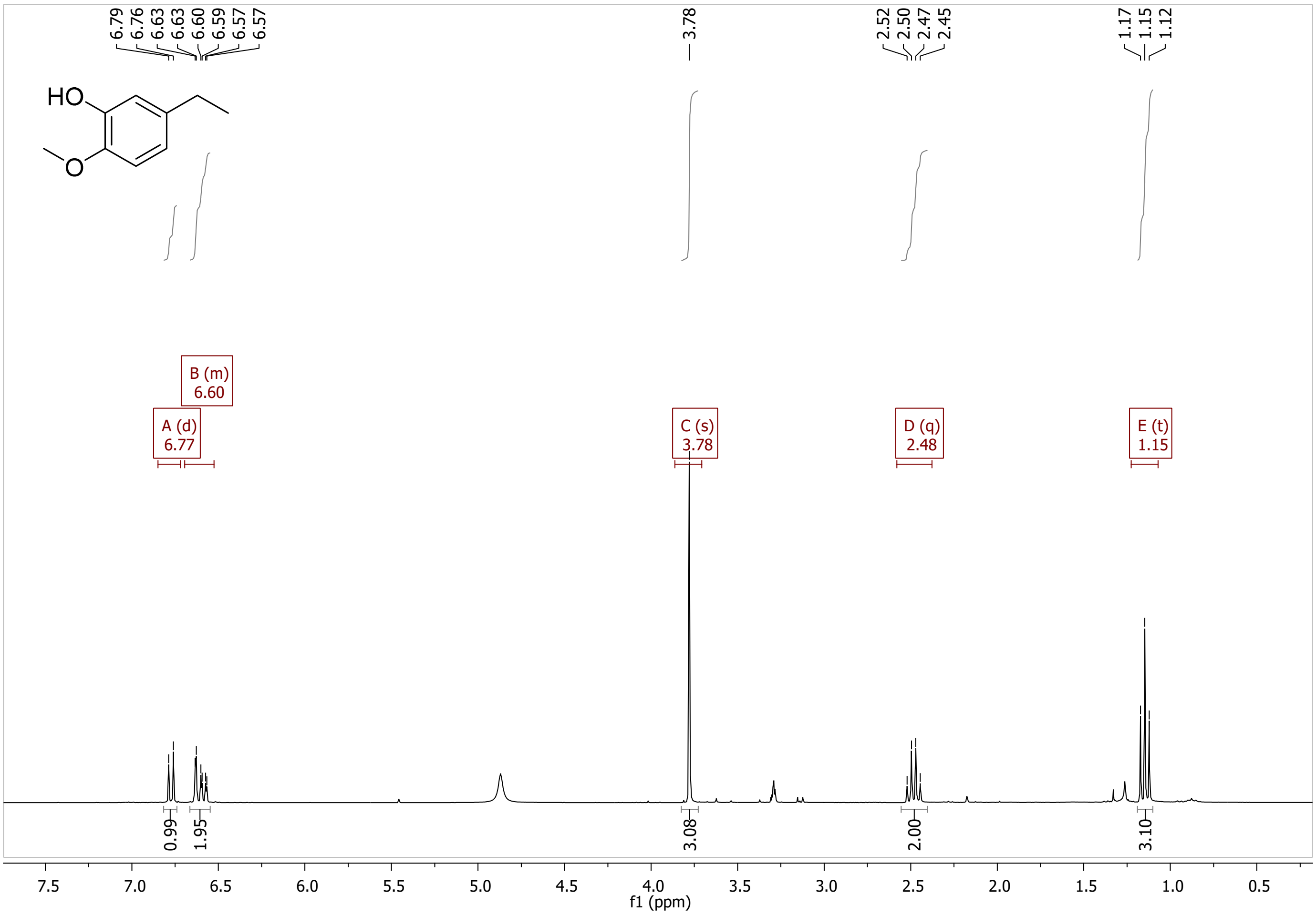

Figure S15. 5-ethyl-2-methoxyphenol *p*-**2h** ^{1}H NMR.

^{1}H NMR (300 MHz, MeOD) δ 6.77 (d, *J* = 8.1 Hz, 1H), 6.69 – 6.52 (m, 2H), 3.78 (s, 3H), 2.48 (q, *J* = 7.6 Hz, 2H), 1.15 (t, *J* = 7.6 Hz, 3H).

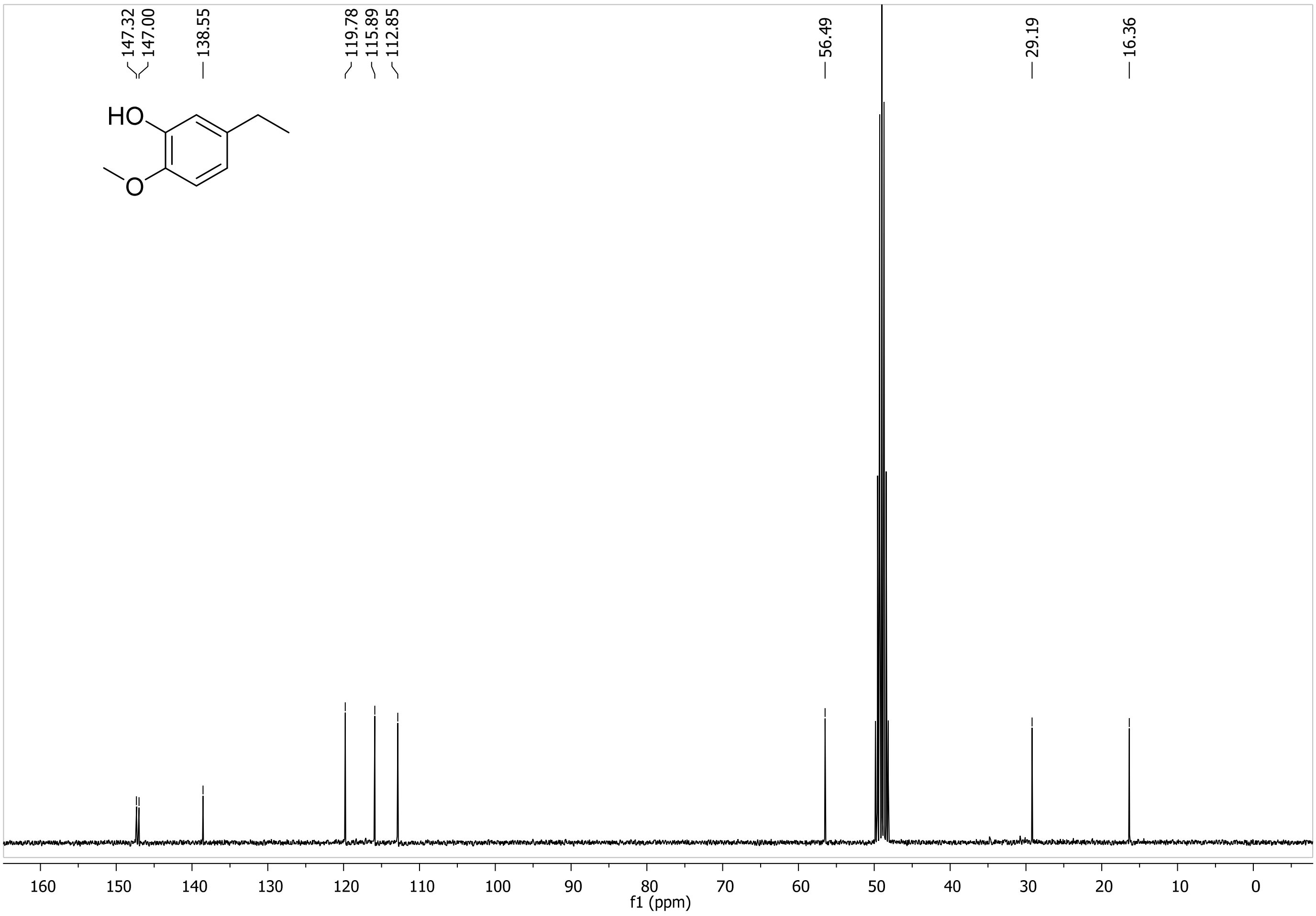

Figure S16. 5-ethyl-2-methoxyphenol *p*-**2h** ^{13}C NMR.
^{13}C NMR (75 MHz, MeOD) δ 147.32, 147.00, 138.55, 119.78, 115.89, 112.85, 56.49, 29.19, 16.36.

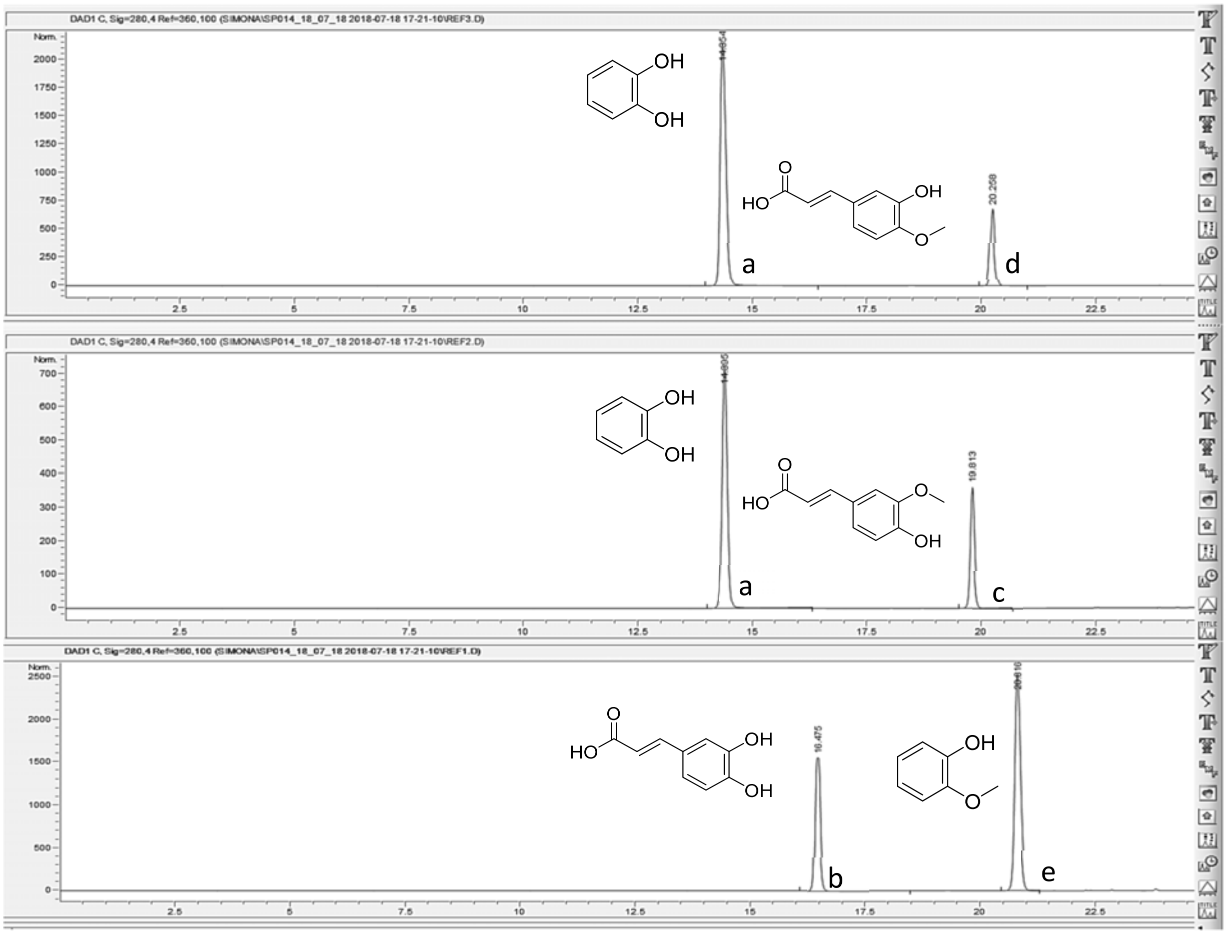

Figure S17. Example of HPLC chromatograms. Commercially available references.

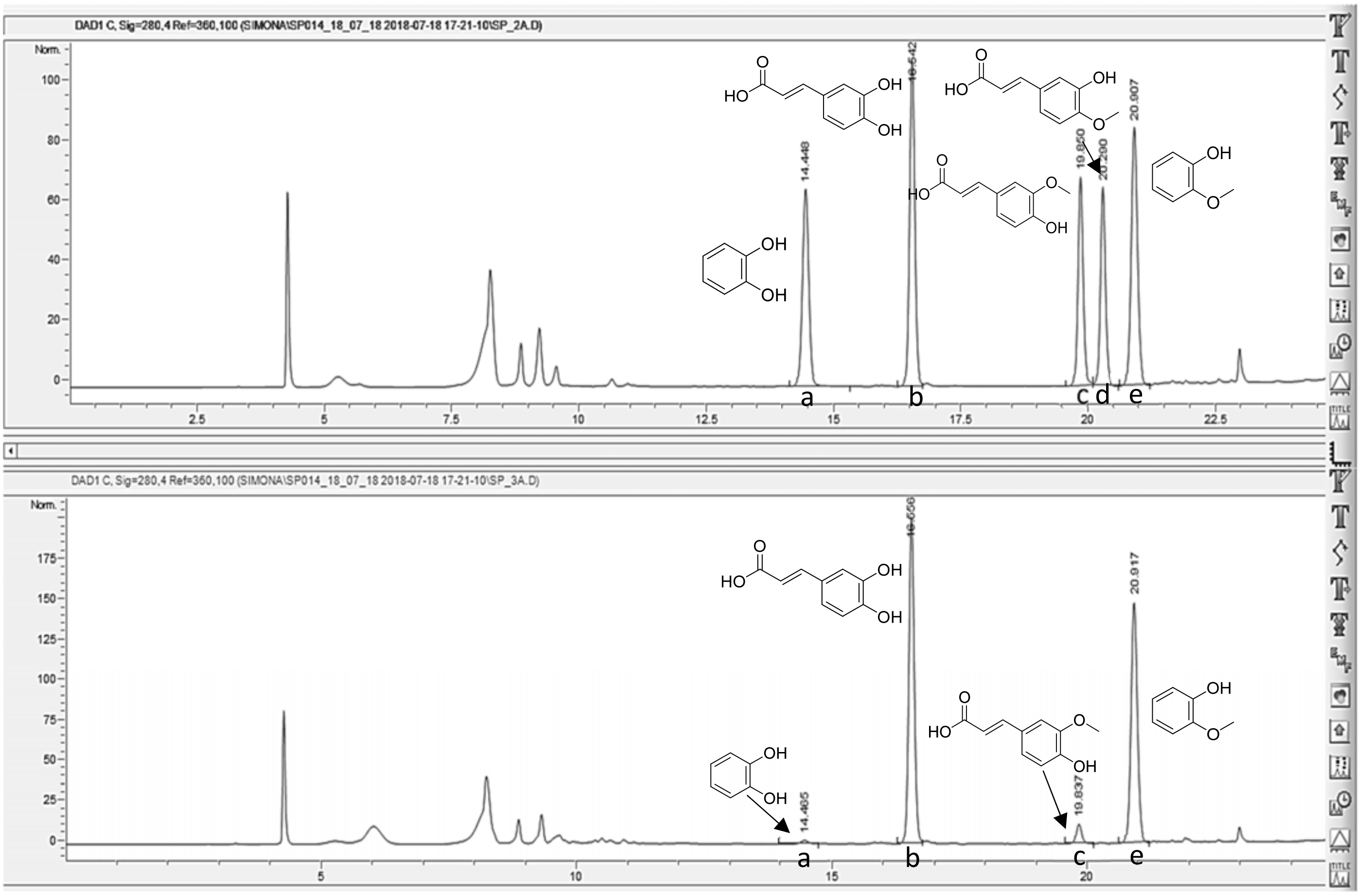

Figure S18. Example of HPLC chromatograms of biotransformations. Top chromatogram: biotransformation in the presence of 10% v/v of MeOH analyzed after 24 h; a) catechol, b) substrate **1b**, c) product *m*-**2b**, d) product *p*-**2b**, e) guaiacol; Bottom chromatogram: biotransformation in the presence of 10% v/v of THF analyzed after 24 h; a) catechol, b) substrate **1b**, c) product *m*-**2b**, e) guaiacol

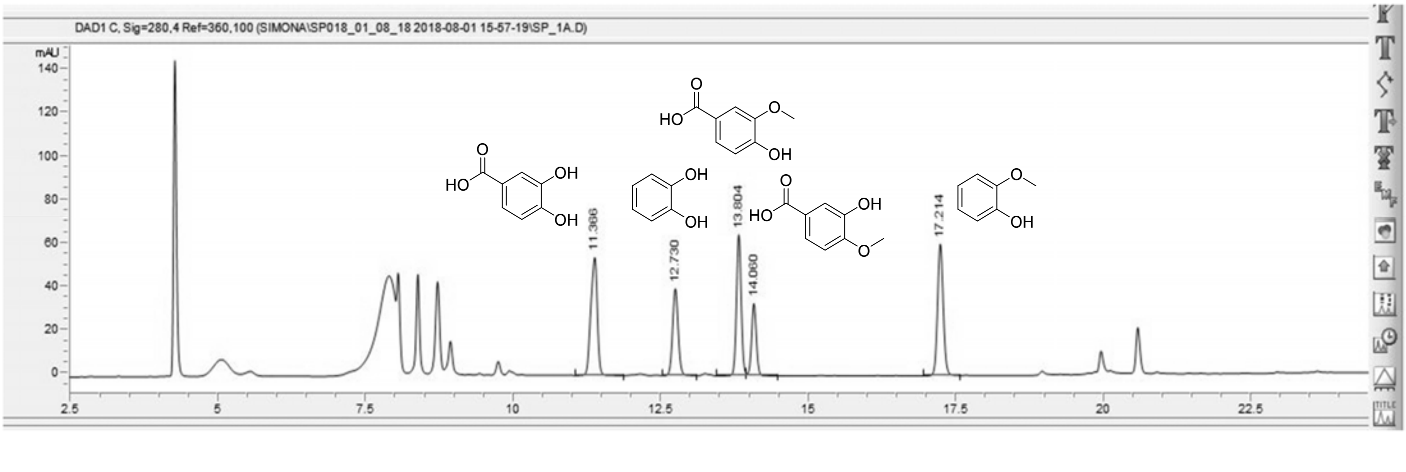

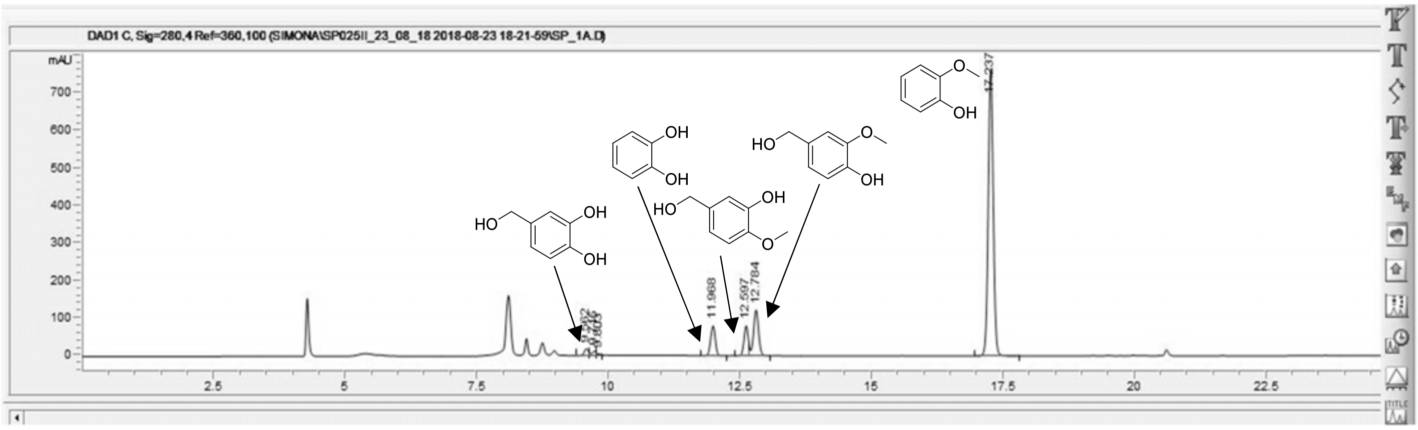
DAD1 C, Sig=280,4 Ref=360,100 (SIMONA\SP025II_23_08_18 2018-08-23 18-21-59\SP_1A.D)
mAU
11.968
12.597
12.784
17.237
OH
HO
O

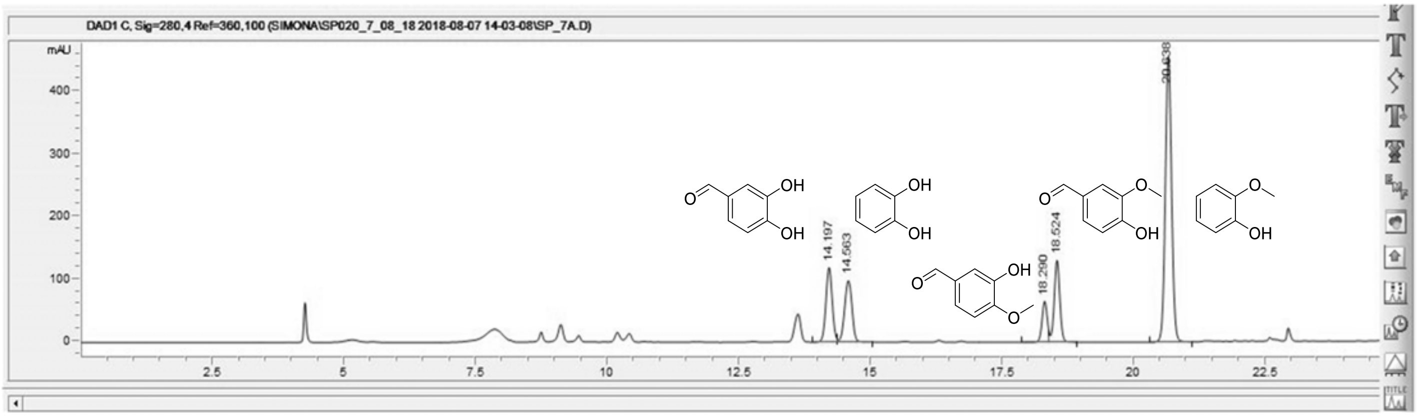
DAD1 C, Sig=280,4 Ref=360,100 (SIMONA\SP020_7_08_18 2018-08-07 14-03-08\SP_7A.D)
mAU
14.197
14.563
18.290
18.524
20.638
OH
O

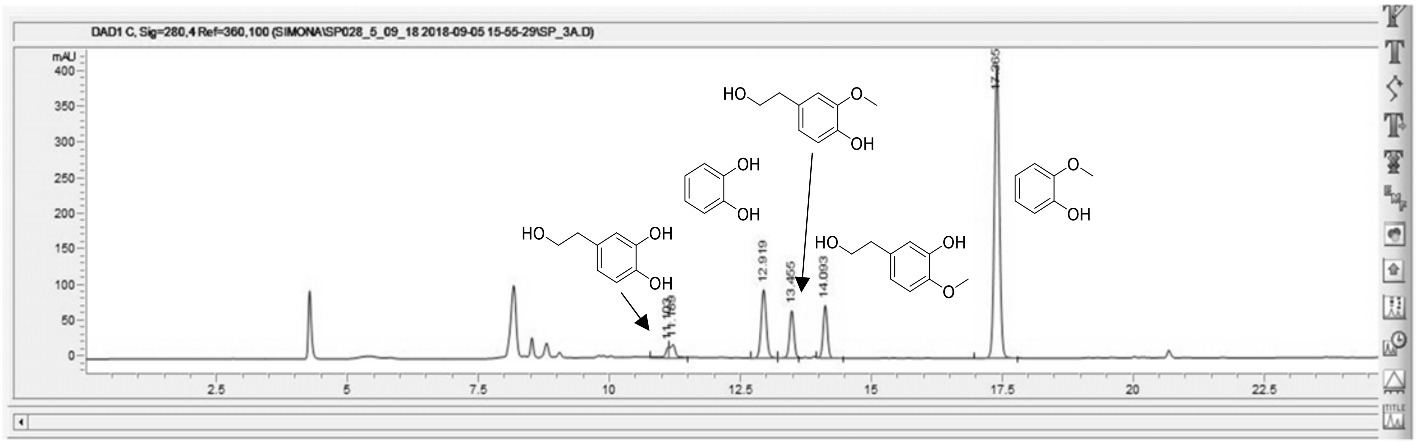
DAD1 C, Sig=280,4 Ref=360,100 (SIMONA\SP028_5_09_18 2018-09-05 15-55-29\SP_3A.D)
mAU
12.919
13.455
14.093
17.365
OH
HO
O

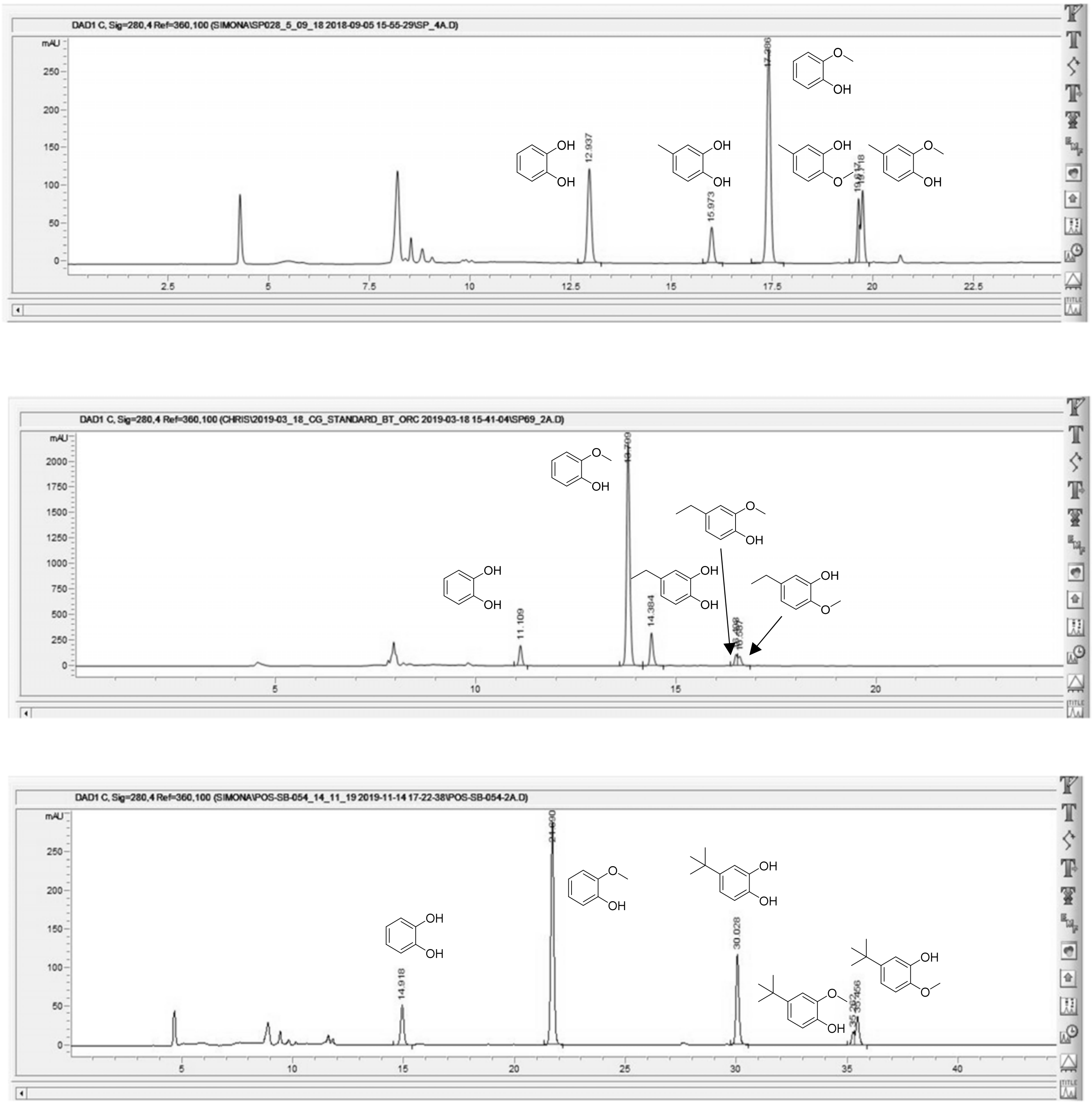

Figure S19. Example of HPLC chromatograms of biotransformations with **XXXYX**.

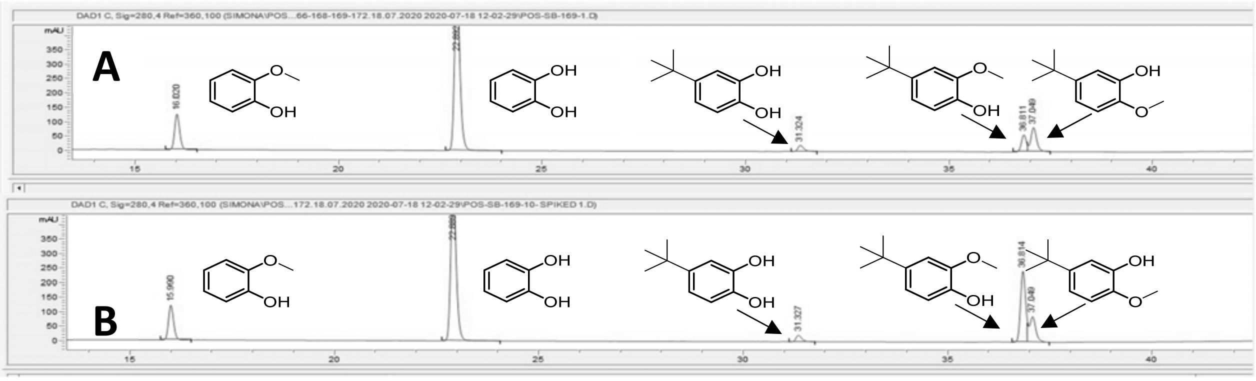

Figure S20. (**A**) HPLC chromatogram of biotransformation and (**B**) spiked chromatogram with the commercial available reference isomer 4-(tert-butyl)-2-methoxyphenol.

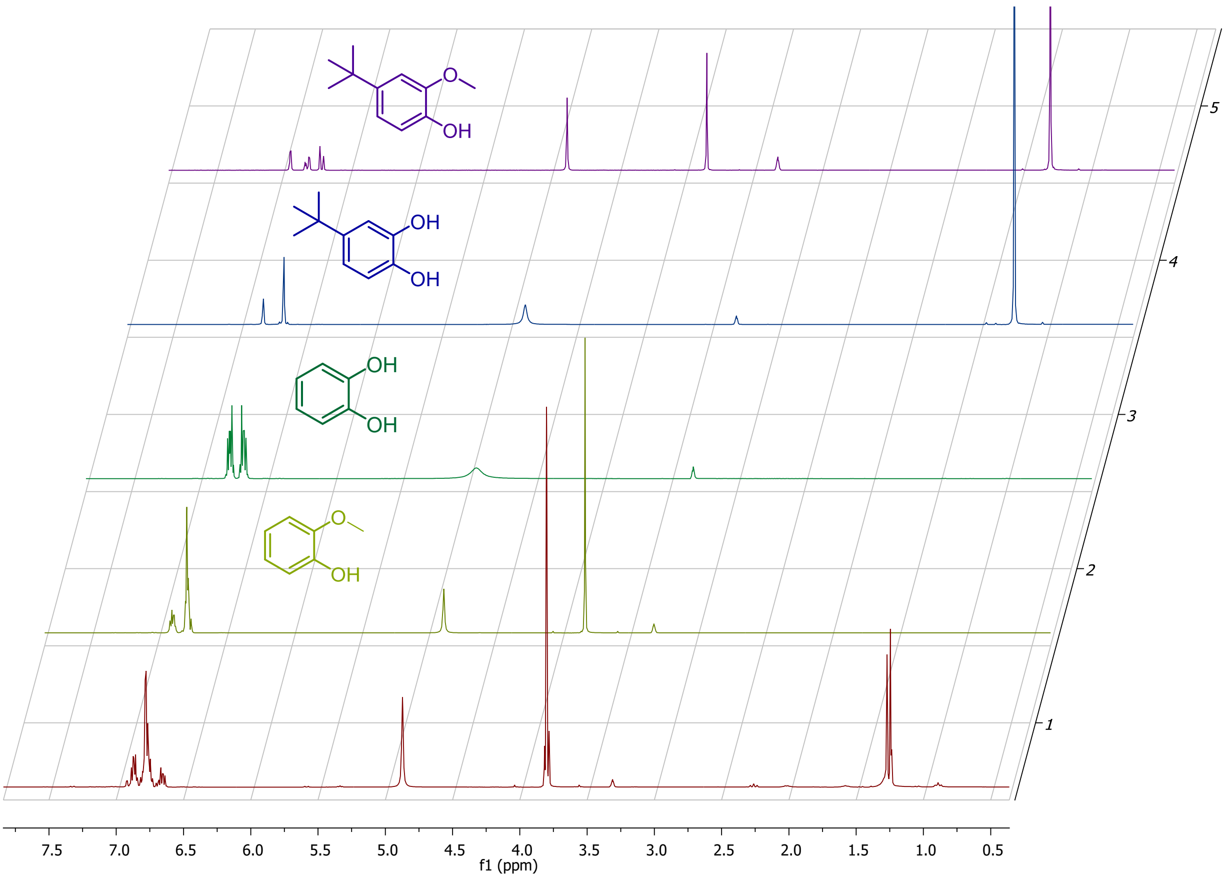

Figure S21. ^{1}H NMR of 24 mL scale methylation of 4-*tert*-butylcatechol. The extracted mixture from the biotransformation is reported in red. The three singlet reported at 3.82, 3.80 and 3.78 ppm belong respectively to 4-(tert-butyl)-2-methoxyphenol, guaiacol and 5-(tert-butyl)-2-methoxyphenol, according to the reference material NMRs (reported in the different colors).

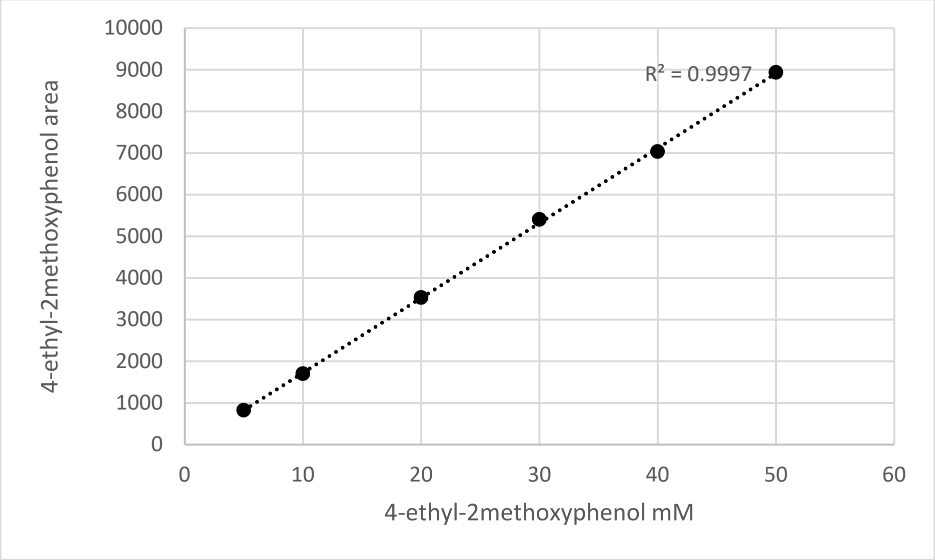

Figure S22. Example of HPLC calibration curve.

Compound	RT [min] method A	RT [min] method B	RT [min] method C	RT [min] method D
Guaiacol	17.7	20.0	14.2	21.9
Catechol	13.3	15.0	11.6	14.1
Vanillic acid	14.2	17.0	n.d.	n.d.
Isovanillic acid	14.5	14.4	n.d.	n.d.
3,4-dihydroxybenzoic acid	11.8	13.5	n.d.	n.d.
Vanillin	15.9	18.2	n.d.	n.d.
Isovanillin	15.7	17.9	n.d.	n.d.
3,4-dihydroxybenzaldehyde	13.2	14.4	n.d.	n.d.
Ferulic acid	16.6	19.6	n.d.	n.d.
Isoferulic acid	16.8	20.1	n.d.	n.d.
Caffeic acid	14.3	16.3	n.d.	n.d.
Vanillyl alcohol	11.8	n.d.	n.d.	n.d.

Isovanillyl alcohol	12.4	n.d.	n.d.	n.d.
3,4-dihydroxybenzyl alcohol	9.6	n.d.	n.d.	n.d.
3-Hydroxytyrosol	10.9	n.d.	n.d.	n.d.
5-(2-hydroxyethyl)-2-methoxyphenol	13.9	n.d.	n.d.	n.d.
Homovanillyl alcohol	12.6	n.d.	n.d.	n.d.
4-methylcatechol	15.9	n.d.	n.d.	n.d.
2-methoxy-5-methylphenol	19.6	n.d.	n.d.	n.d.
2-methoxy-4-methylphenol	19.7	n.d.	n.d.	n.d.
4-ethylcatechol	18.7	21.9	14.4	n.d.
2-methoxy-5-ethylphenol	20.7	n.d.	16.6	n.d.
2-methoxy-4-ethylphenol	20.8	n.d.	16.5	n.d.
4-*t*butylcatechol	20.5	22.9	n.d.	29.6
2-methoxy-5-*t*butylphenol	n.d.	n.d.	n.d.	35.1
2-methoxy-4-tbutylphenol	n.d.	n.d.	n.d.	34.9

Table S4. HPLC retention times and used methods for the screened substrates and products.

References

[1] A. Gambacorta, D. Tofani, R. Bernini, A. Migliorini, *J. Agr. Food. Chem.* **2007**, *55*, 3386-3391.
